# Supplementary material for: A new two-fingered dinosaur sheds light on the radiation of Oviraptorosauria
Source: R Soc Open Sci. 2020 Oct 7;7(10):201184. doi: 10.1098/rsos.201184 (PMC7657903; doi:10.1098/rsos.201184)
Supplement: Supplementary Information [file rsos201184supp1.pdf]

Supplementary Information

A new two-fingered dinosaur sheds light on the radiation of Oviraptorosauria

Funston, Gregory F., Chinzorig, Tsogtbaatar, Tsogtbaatar, Khishigjav, Kobayashi, Yoshitsugu, Sullivan, Corwin, Currie, Philip J.

Contents

1. Expanded Diagnosis
2. Histological Results and Age Estimation
3. Expanded Statistical Methods
4. Phylogenetic Results
5. History of the Specimens
6. Referral of Specimens
7. Provenance of the Poached Specimens
8. Taphonomy of the Holotype
9. Table of age ranges
10. Measurements of *Oksoko avarsan*
11. Character List
12. Character States of *Oksoko avarsan*
13. Supplementary References
14. Supplementary Figures

1. Expanded Diagnosis

Oksoko avarsan can be distinguished from citipatiine oviraptorids by the enlarged first manual digit and reduced second and third manual digits. It can be distinguished from most heyuanniine oviraptorids by the presence of a cranial crest (Fig. S1). Two heyuanniines are known which possess a cranial crest: *Nemegtomaia barsboldi* and *Banji long*. In both of these taxa, the cranial crest is composed primarily of the nasals and premaxilla, whereas in *Oksoko avarsan* the rounded, domed crest is composed primarily of the nasals and frontals.

Two other oviraptorids possess similar cranial crests: *Rinchenia mongoliensis* and *Corythoraptor jacobsi*, both of which are currently considered citipatiine oviraptorids. The skull of *Oksoko avarsan* can be distinguished from *Rinchenia mongoliensis* [1] by the position of the naris dorsal to the orbit; a proportionally greater contribution of the frontal to the cranial crest; a longer tomial part of the premaxilla; a relatively smaller infratemporal fenestra; and a non-interfingering contact between the jugal and quadratojugal (Fig. S2). Postcranially, *Oksoko avarsan* differs from *Rinchenia mongoliensis* in possessing a posteriorly concave axis; elongate, rather than plate-like distal chevrons (Fig. S3); a more strongly everted acromion process of the scapula; and a less dorsoventrally expanded ilium with an accessory brevis ridge (Fig. S4). Unfortunately, the forelimb of *Rinchenia* is incompletely known, and its manus cannot be compared to that of *Oksoko*. *Oksoko avarsan* can be distinguished from *Corythoraptor jacobsi* [2] by a convex, rather than concave anterior surface of the premaxilla; an ovoid, rather than slit-like naris; a relatively shorter neck composed of procoelous cervical vertebrae with large epipophyses; a reduced forelimb with antebrachium subequal to humerus; and by its short manus with an enlarged first digit and vestigial third digit. These features are consistent between the ontogenetic stages of *Oksoko avarsan*, and it is unlikely that the radical differences in the skull or forelimb are the result of individual variation. Little is known about ontogenetic changes in the crests of oviraptorosaurs, but evidence from juvenile *Conchoraptor gracilis*[3] and embryonic *Citipati osmolskae*[4] suggest that the presence of a crest and its constituent bones are consistent throughout ontogeny. Norell et al.[4] first suggested that crests may have been ontogenetic because they were as yet unknown in small oviraptorids. The presence of well-developed cranial crests in clearly juvenile *Oksoko avarsan* fills this gap and, alongside the small, crested *Banji long*, argues against ontogenetic development of crests in oviraptorids.

2. Histological Results and Age Estimation

MPC-D 102/11—The femur and fibula differ in the degrees of secondary remodeling, but both lack any lines of arrested growth. The femur (Fig. S5) is entirely composed of primary fibrolamellar bone with no secondary osteons. Vasculature is primarily arranged longitudinally, although there are some regions of reticular vasculature. A zone of parallel-fibred bone occurs towards the periosteal surface of the bone. This zone is similar to a growth mark described in a caenagnathid tibia from the Horseshoe Canyon Formation of Alberta[5], and likely represents the same phenomenon. The fibula has considerably more secondary remodeling than the femur, but it is still predominantly primary bone. On the lateral side, the cortex is mostly remodeled, except for a region of primary bone near the periosteal surface that has numerous Sharpey's

fibers (Fig. S6a). On the medial side, there is primary osteonal bone towards the endosteal surface and avascular parallel-fibred bone near the periosteal surface. The medullary cavity and endosteal lamellae are both well developed. Like the femur, there is an annulus developed towards the periosteal surface that probably represents the first growth mark. Based on these data, MPC-D 102/11 is best regarded as a young juvenile approximately one year of age.

MPC-D 102/110.a—The fibula (Supplementary Fig. S5) is composed mostly of primary fibrolamellar bone, but there are some secondary osteons on the medial side. There is a high proportion of woven bone with dense, plump osteocyte lacunae (Fig. S6b). The medullary cavity is small but has defined edges and in some places, there are endosteal lamellae. There are no obvious zones of parallel-fibred bone or lines of arrested growth. Towards the periosteal surface on the anterior side of the fibula there is a region of reduced vasculature, but no obvious annulus is present and this zone cannot be traced around the entire cortex. This may be incipient development of the annulus recorded in the other individuals. It is unlikely that a growth mark has been obscured by secondary remodeling because secondary osteons are sparse and primary bone is visible between them. However, the sampling location may have affected the preservation of growth marks. The predominance of primary bone and absence of a growth mark suggest this individual is a young juvenile, but no age assessment can be made.

MPC-D 102/110.b—Two fragments of the fibula (Fig. S5) were sectioned and both have similar histological texture. Neither fragment shows the medullary cavity, but this may be because the samples were taken distally. Both are composed predominantly of primary fibrolamellar bone, but each has some secondary osteons endosteally. Vasculature is mostly longitudinal, but there are some small zones of reticular canals. Near the periosteal surface there is a zone of avascular, parallel-fibred bone similar to that in MPC-D 102/11. It likely represents the first growth mark, although no distinct rest line is visible. Like MPC-D 102/11, this specimen was likely a young juvenile approximately one year of age.

MPC-D 102/12—The fibula (Fig. S5) and a fragment of the femur were sectioned. The fibula has more secondary remodeling, and therefore some of the growth record has been erased (Fig. S6c). The medullary cavity is well formed and lined by multiple generations of endosteal lamellae. Several other erosive cavities also excavate the cortex; these are separated by trabeculae or endosteal lamellae. Vasculature is longitudinally oriented, and towards the medial surface these canals are arranged into circumferential rows. At least three lines of arrested growth are recorded on the medial surface, but it is likely that more have been obscured by secondary remodeling and expansion of the medullary cavity.

The femur exhibits less secondary remodeling, all of which is concentrated in a vertical column extending perpendicular to the periosteal surface (Fig. S6d). The primary bone in this column contains larger, more densely packed osteocyte lacunae, and well-developed Sharpey's fibers towards the periosteal surface (Fig. S6d). Accordingly, it likely represents a zone of muscle insertion. A small strip of endosteal lamellae marks the edge of the medullary cavity, indicating that the entire cortex is preserved. Vasculature changes from predominantly reticular endosteally through plexiform towards a zone of parallel-fibred bone about halfway through the cortex. A faint cement line is visible within this zone of parallel-fibred bone, indicating that it represents a growth mark in the

form of an annulus. External to this annulus, vasculature is laminar and decreases towards the periosteal surface. At least four more annuli are visible in the external part of the cortex, and have decreased spacing periosteally. Whereas a zone of fibrolamellar osteonal bone separates the first, second, and third annuli, the spaces between the third, fourth, and fifth annuli are composed of parallel-fibred bone. This forms a continuous band of parallel-fibred bone on the periosteal surface of the cortex. Although no distinct lines of arrested growth can be distinguished in this area, this is likely the result of the light colour of the bone and the reduced thickness of the slide. In any case, the dominance of parallel-fibred bone at the periosteal surface indicates that this individual was growing slowly[6–10]. The presence of five annuli and the low growth rate suggest that this individual was an adult at least five years old and was approaching maximum body size.

3. Expanded Statistical Methods

To trace the patterns of digit and limb reduction in oviraptorids, a proxy for digit size was developed. We follow the conventional nomenclature for manual digits of digits I, II, and III, because those are the phenotypes expressed, although we realize these may be homologous to digits II, III, and IV [11]. Measurements for 73 complete digits (digit I, n = 32; digit II, n = 29; digit III, n = 12) of 31 oviraptorosaur specimens (15 taxa) showed that straight-line length of the ungual was strongly correlated to the pre-ungual length of that digit ($0.81<R^2>0.88$; Fig. S7). Therefore, the ratio of two ungual lengths could be used to determine the relative proportions of two digits, even where those digits were not complete. Based on these correlations, the ratio of ungual III-4 to I-2 was used because it maximized the availability of data: 23 of 39 oviraptorosaur taxa (totaling 29 specimens) have both ungual I-2 and III-4, whereas only 9 of more than 100 measured oviraptorosaur specimens have complete first and third digits. To improve estimation of the root condition for maximum likelihood analysis, additional outgroups representing a broader array of coelurosaurs were grafted to the majority-rules phylogeny (following the topology of Hendrickx et al.[12]) to improve estimation of the root condition (Figs. S8, 9). *Archaeopteryx* was removed as an outgroup because its forelimb and manual proportions are unlikely to be representative of the basal coelurosaurian condition. This was necessary because the forelimbs of *Incisivosaurus* are not known, which overweighed the influence of *Archaeopteryx* on the root condition.

Forelimb length for oviraptorosaurs was calculated as the sum of the length of the humerus, ulna, and metacarpal II, but not including the lengths of the digits. Forelimb length was divided by femoral length and mapped as a continuous character in the same way as the ratio of ungual III-4 to I-2. Allometry of the forelimb in oviraptorosaurs was tested using bivariate plots against log-transformed femur length (Main Text: Fig. 5).

In addition to mapping the ratio of ungual III-4 to I-2 on a phylogeny, the relative proportions of the digits for 17 oviraptorosaurs (cyan) with complete hands or scaled composites and 67 theropods (grey) were plotted in a ternary plot (Main Text: Fig. 5). Scaled composites were created by scaling overlapping elements to the same length to estimate missing measurements where a missing element is known from another side or another specimen. Composites were created in this way for *Chirostenotes*, *Nemegtomaia*, and *Oviraptor*. Digit length, including the ungual, was calculated from straight-line

measurements of the long axes of each phalanx. The length of each digit was divided by the sum of the lengths of all digits and plotted as a percentage.

The history of oviraptorosaur biogeography was stochastically simulated based on the tip data for 1000 replicates using the make.simmap function of *phytools* v0.6-44, using a continuous-time reversible Markov model with equal rates of transformation. This allowed for estimation of ancestral biogeographic states and the posterior probability of each state at each node. This model is necessarily simplistic, because of the limited information on the stratigraphy and geographic ranges of each taxon. In the future, it may be possible to increase the precision of the Markov model by scaling transformation rates based on biogeographic information, but this was not feasible for the present study. The results of the analysis were confirmed by S-DIVA analysis in RASP, which allows nodes and tips to be present in more than one area.

4. Phylogenetic Methods and Results

Methods

Matrix construction—A morphological character matrix for maximum parsimony was compiled based on the previous analyses of Osmólska et al.[13], Lamanna et al.[14], Funston and Currie[15] and Lü et al.[2]. The tree was modified from these previous analyses mostly by dropping or consolidating caenagnathid taxa. In particular, *"Macrophalangia canadensis*" and *"Caenagnathus sternbergi*" were subsumed into *Chirostenotes pergracilis*, which was updated based on new specimens[1]. Characters scored for "Alberta Dentary Morph 3" were added to *Citipes elegans*, and the former operational taxonomic unit (OTU) was removed. *Ojoraptorsaurus boerei* was also removed because it could be coded for relatively few characters (approximately 1%). *Leptorhynchos gaddisi* was removed because it is represented only by the mandible and is therefore provides little data. *Oksoko avarsan* was added to the matrix and could be coded for nearly all of the characters (97.5%). Despite their early ontogenetic stages, *Banji long*, *Microvenator celer*, and *Yulong mini* were included in the analysis. Juvenile OTUs are typically recovered more basal than they should be, but this can be corrected by not scoring ontogenetically variable characters for these OTUs[16]. Although caenagnathid ontogeny is more poorly known, analyses of oviraptorid ontogeny (G.F.F., P.J.C. pers. obs.) indicate that few—if any—characters in the matrix are ontogenetically variable. Accordingly, these juvenile OTUs are likely to provide at least some important information.

Several characters were dropped from previous analyses because they were uninformative, ontogenetically variable, or poorly constructed. Character 110 of Lü et al.[2], regarding the state of cervical rib fusion, was dropped because it varies through ontogeny. Character 215 on the position of muscle scars on the dorsal vertebrae was removed because it provided little information and the positions can change throughout both ontogeny and the axial series. Most of the characters removed were those added by Funston and Currie[15], because these were poorly constructed and overweighted the influence of the manual phalanges and metatarsus. These characters were initially introduced to provide more information on the relationships of caenagnathids, but improved sampling of the taxa accomplished the same task more rigorously. In addition to removing characters, all of the characters were treated as unordered. Previously ordered characters are highlighted in the Character List, but because they applied only to

a few taxa, they served to force relationships artificially and were removed for this analysis.

Three outgroups were included in the anaylsis: *Herrerasaurus ischigualastensis*, *Velociraptor mongoliensis*, and *Archaeopteryx lithographica*. Besides being the taxa already used by the matrix of Osmólska et al.[13], these outgroups are appropriate because they polarize primitive characters within both Saurischia and Coelurosauria. *Herrerasaurus* is typically recovered as a basal saurischian or theropod, and therefore provides information on the ancestral characters of all theropods. *Velociraptor* and *Archaeopteryx* were both included in the matrix of Maryañska et al.[17] and Osmólska et al.[13] to test whether oviraptorosaurs were more closely related to birds than other theropods. Nonetheless, they each provide an appropriate polarization for paravian characters.

The resulting matrix had 42 taxa and 246 characters, which were a mix of binary and multistate characters. All multistate characters were treated as unordered. The matrix has a relatively high proportion of missing data: 51.7% of the characters could not be coded. As expected, much of this poor data quality comes from the caenagnathid portion of the tree, where only about one-third of the characters could be coded (68.7% missing data). This likely accounts for their volatility in previous analyses, typically resulting in a 13-tomy of caenagnathids more derived than *Gigantoraptor erlianensis.* Regardless, this is still a drastic improvement over previous analyses—for example, the caenagnathid portion of the matrix of Lü et al.[2] has 80.6% missing data. Oviraptorids, in contrast, could be coded for more than half of the characters (46.0% missing data) and have been relatively stable in most analyses. However, some analyses have differed in the membership of each subfamily, and therefore better resolution in the oviraptorid portion of the tree is still important.

Tree search—A parsimony-based heuristic tree-search was run in TNT v.1.1 using 10000 replications of Wagner trees followed by tree bisection-reconnection (TBR) branch swapping, holding up to 10 trees each replication. A final round of TBR branch swapping was used on the resulting trees to find additional most parsimonious trees. Bremer support values were calculated using the *Bremer.run* package included with TNT v1.1. The analysis produced nine most parsimonious trees of 641 steps, with relatively strong Bremer support for the major clades of Oviraptorosauria. The strict consensus tree (CI: 0.41, RI: 0.63) has a polytomy at the base of Oviraptorosauria within Caudipteridae, and a second polytomy within Oviraptoridae between non-heyuannine oviraptorids. Otherwise, the phylogeny is completely resolved. The majority-rules consensus tree (CI: 0.43, RI: 0.66) is completely resolved and was therefore used for the subsequent analyses.

Statistical methods—The phylogeny was time-scaled using age ranges published from the literature. Although the ages of most taxa could be determined relatively precisely, the stratigraphic ranges of oviraptorids from southern China are poorly constrained. In these cases, stratigraphic ranges were taken from published estimates of the ages of the formations where the specimens were found. Time-scaling was done using the *paleotree* v3.3.0 package in R statistical package. Branch lengths were calculated using the equal dating method of Brusatte et al.[18].

Results

The cladistic analysis produced nine most parsimonious trees of 641 steps (all trees are available in the included Nexus file). The strict consensus tree has polytomies near the base of Oviraptorosauria and within oviraptorids, but is otherwise well resolved (Fig. S10). The majority-rules tree is completely dichotomous (Figs. S8, 9), and this exact topology is also recovered among the most parsimonious trees (Tree 3 of the supplementary Nexus file). Although the majority-rules tree does not inherently add or resolve information to the analysis, most downstream analyses require fully dichotomous trees. This topology was chosen for subsequent analyses, rather than randomly resolving dichotomies, because this topology is among the most parsimonious trees. In other words, it is a well-supported choice for a preferred topology.

Incisivosaurus gauthieri is recovered as the earliest-diverging oviraptorosaur, followed by a paraphyletic Caudipteridae where *Similicaudipteryx yixianensis* is an outgroup to the sister taxa *Caudipteryx dongi* and *Caudipteryx zoui*. These species of *Caudipteryx* are sisters to a well-supported (decay index 2) group of *Avimimus* and Caenagnathoidea, referred to here as Edentoraptora based on the ubiquitous absence of teeth in these animals. The species of *Avimimus* are recovered as sister taxa to each other and together are sister to Caenagnathoidea. Caenagnathoidea is well-supported (decay index 3) and is divided at its base into Caenagnathidae and Oviraptoridae.

The most primitive caenagnathid is *Microvenator celer*, followed by *Gigantoraptor erlianensis*, which is sister to the so-called 'derived caenagnathids'. These taxa are characterized by relatively small size and complexly textured occlusal surfaces of the fused dentaries. This group is relatively well-supported (decay index 2), and therefore referred to here as Caenagnathinae for clarity. The most primitive caengnathines are the sister taxa *Chirostenotes pergracilis* and *Hagryphus giganteus*. The recovery of the latter taxon as sister to *Hagryphus giganteus* is based on manual characters, and their basal position is novel, but not unusual considering the unusual morphology of the pelvis of *Chirostenotes pergracilis* compared to more derived caenagnathids like *Anzu wyliei*, *Caenagnathus collinsi*, *Citipes elegans*, and *Nomingia gobiensis*. The latter taxon, *Nomingia gobiensis*, is recovered between *Hagryphus giganteus* + *Chirostenotes pergracilis* and a clade of the other caenagnathines, rather than as the sister to *Elmisaurus rarus*, which provides some support for its taxonomic distinction. *Nomingia gobiensis* has typically been recovered as an oviraptorid on the basis of the rounded preacetabular blade and the astragalus reaching the lateral margin of the tarsus. However, the discovery of these features in other caenagnathids draws it to the caenagnathid portion of the tree, which is more appropriate based on the similarity of the rest of its skeleton to caenagnathids[19]. *Elmisaurus rarus* and *Citipes elegans* are recovered as sister taxa, which is unsurprising considering the distinctiveness of their fused metatarsi. Their position within Caenagnathinae obviates the need for the name 'Elmisaurinae', although if future representatives of this lineage are discovered, Elmisaurini may be appropriate. *Apatoraptor pennatus* is the sister taxon to a dichotomous pair of sister taxa, *Caenagnathasia martinsoni* and *Epichirostenotes curriei*, as well as *Anzu wyliei* and *Caenagnathus collinsi*. The sister relationship of *Caenagnathasia martinsoni* and *Epichirostenotes curriei* is somewhat surprising, considering the disparate ages and body sizes of these taxa. However, there is some likelihood that the material referred to *Caenagnathasia martinsoni* by Sues and Averianov [20] makes it a chimaera, because it combines a suite of relatively primitive

oviraptorosaur postcranial features with derived features of the mandible. Furthermore, a specimen of *Caenagnathasia martinsoni* that bore teeth was allegedly collected (J. Stiegler pers. comm.), but was lost in transit. If this is true, it provides strong evidence that *Caenagnathasia* is not an edentoraptoran, but rather a late-surviving early oviraptorosaur that converged on the complex dentaries of caenagnathids. There is some support for this in the differing arrangement of the occlusal structures of *Caenagnathasia martinsoni* from other caenagnathines, the large anterior occlusal groove, and the lack of lateral occlusal ridges. The union of *Anzu wyliei* and *Caenagnathus collinsi* is unsurprising based on their mandibles.

Oviraptoridae consists of *Nankangia jiangxiensis, Oviraptor philoceratops*, and *Yulong mini* as successive outgroups to two subfamilies of oviraptorids. The basal position of *Yulong mini* is likely the result of its early ontogenetic stage. The oviraptorid subfamilies appear to be divided based on the morphology of the manus. One of the two groups is comprised of forms with elongate manus with digits roughly equal in robustness and an elongate third digit. Based on the exclusion of *Oviraptor philoceratops* from this group, it is inappropriate to refer to this family as 'Oviraptorinae', as in previous studies. Instead, it is hereby designated Citipatiinae because *Citipati* is the oldest valid genus named in the family[21]. Although *Rinchenia* was coined earlier[22], this genus was not properly diagnosed until its type species was synonymized with *"Oviraptor" mongoliensis* in 2004[13].The most primitive citipatiine is *Wulatelong gobiensis*, and this is followed by a sister clade of *Rinchenia mongoliensis* and *Tongtianlong limosus*. *Ganzhousaurus nankangensis* is the sister to a group of *Citipati osmolskae* and the Dzamyn Khondt oviraptorid as sister taxa and *Corythoraptor jacobsi* and *Huanansaurus ganzhouensis* as sister taxa. Notably, Citipatiinae includes nearly all of the oviraptorids from southern China. The other group of oviraptorids has been previously referred to as 'Ingeniinae', but because '*Ingenia*' is preoccupied, this subfamily name is inappropriate. *'Ingenia' yanshini* was previously incorporated into *Heyuannia* as *Heyuannia yanshini*[1], and so this genus now lends its name to the subfamily: Heyuanninae. *Shixxingia oblita* is recovered as the most basal heyuannine, but this taxon is poorly known and it is volatile in the phylogeny. *Khaan mckennai*, *Conchoraptor gracilis*, and *Machairasaurus leptonychus* are successive outgroups to the remaining heyuannines, which form two sister clades of three taxa each. *Nemegtomaia barsboldi* is sister to the two species of *Heyuannia* on one side, and *Banji long* is sister to a clade of *Jiangxisaurus ganzhouensis* and *Oksoko avarsan* on the other side.

Notably, these trees differ from recent analyses[2,23] in the positions of certain Chinese oviraptorids. Lü et al.[2] recover a clade of *Tongtianlong*, *Wulatelong*, and *Banji* at the base of Heyuanniinae. Our analysis divides this clade, placing *Banji* as a derived heyuaniine and *Tongtianlong* and *Wulatelong* as sister taxa in a clade of citipatiines. *Ganzhousaurus*, recovered as a derived heyuanniine by Lü et al.[2], is also placed within this new citipatiine clade. The result is a group of closely related but anatomically disparate southern Chinese oviraptorids, which supports recent proposals that oviraptorids radiated in southern China in the Campanian–Maastrichtian. Furthermore, it provides evidence that at least part of this diversification may have been a *bona fide* adaptive radiation, as suggested by previous work[23].

5. History of the Specimens

To date, four specimens totaling seven individuals of *Oksoko avarsan* are known. The first known specimen of this taxon (MPC-D 100/33) was collected by the 1974 Soviet-Mongolian Palaeontological Expedition at Bügiin Tsav. The specimen is housed at the Institute of Paleontology and Geology in Ulaanbaatar, Mongolia, and, at the time of writing, is on display at Hunnu Mall in the southwestern part of the city.

Two poached specimens provide most of the anatomical information. MPC-D 102/110, the holotype, is an assemblage of 3 individuals crouched in life positions. MPC-D 102/11-A is a partial skeleton of a young individual, including a skull, anterior cervical vertebrae, pelvis, hindlimbs, and tail. Included with this specimen is a large quadrate and quadratojugal (MPC-D 102/11-B), which are slightly larger than the partial skeleton. The skull of the partial skeleton has both quadrates and quadratojugals, so the isolated elements must be from a different individual. The morphology of the quadrate and quadratojugal—and their preservation—is identical to the partial skeleton and the individuals in MPC-D 102/110, indicating that they are from the same taxon and likely same assemblage. MPC-D 102/12 is a postcranial specimen of an adult individual, as indicated by histology showing slowed growth and the extensive fusion of the bones.

MPC-D 102/110 and MPC-D 102/11 were confiscated by The General Intelligence Agency of Mongolia sometime in 2006, and were both returned to the MPC December 14th, 2006. Both specimens were prepared at the MPC. Thus, neither the positions of the specimens nor their anatomy could have been modified by the poachers, and their current positions reflect their postures upon death, burial, and excavation. MPC-D 102/11 was prepared by G. Enkhtuul, and the specimen was mostly disarticulated during preparation. Unfortunately, no photographs were taken prior to or during preparation. Preparation on MPC-D 102/110 was undertaken by Ch. Bayardorj and involved stabilizing the poorly-made field jacket, which was too thin and included plastic shopping bags as a separating layer. The fragile nature of the specimen and the association of the skeletons means the specimens were not disarticulated. Some photos were taken by the last author during preparation in 2007, which confirm that the specimens were not artificially associated (Fig. S11). Additional preparation and disarticulation of the left hand of MPC-D 102/110-A was carried out by the lead author. All three hands preserved in the block were fully encased in matrix when prepared, and therefore the phalanges of the third digit could not have gone missing through erosion or during collection. The preservation of delicate structures like the scleral ossicles and complete articulation of the skeletons suggests that the distal phalanges are not absent because of disarticulation or transport before burial. The provenance of MPC-D 102/11 and MPC-D 102/110 were not recorded when the specimens were returned to the MPC, but were determined using geochemical approaches[24] (see section on 'Provenance').

MPC-D 102/11 shares many taphonomic features with MPC-D 102/110, including the crouched posture (Fig. S12), the nature of the remaining matrix, and the fine quality of preservation. Furthermore, it is unlikely that they were returned on the same date coincidentally. The specimens were probably excavated by and confiscated from the same poachers, and were likely collected from the same deposit or, at least, nearby sites. It is likely that the extraordinary postures of both specimens are the result of

the same depositional circumstances, which are unusual for the Nemegt Formation, where articulated specimens are usually in opisthotonic death poses, laying on their sides, with the necks and tails arched backwards. It is therefore likely that MPC-D 102/11 is part of the same assemblage as MPC-D 102/110. Were this the case, it is worthy of note that the isolated quadrate and quadratojugal of MPC-D 102/11 are approximately the same size as those of the individuals in MPC-D 102/110.

MPC-D 102/12 was collected in 1998 by a joint expedition from the Hayashibara Museum of Natural Sciences and the Mongolian Palaeontological Center[25]. It was discovered at Guriliin Tsav by Ch. Bayardorj, and GPS data was recorded. It is from geographically close to Bügiin Tsav, which geochemical data suggest is the provenance of the repatriated specimens. It therefore supports this interpretation and provides a concrete stratum for the taxon. Manual ungual I-2 was recovered in 2018 when the site was revisited.

6. Referral of Specimens

Besides the holotype block, three other specimens of *Oksoko avarsan* are known (Fig. S13). Numerous characters justify the referral of the paratypes to *Oksoko avarsan*. MPC-D 100/33 was initially included as a paratype of *'Ingenia' yanshini*[26,27] (now *Heyuannia yanshini*)[1], which created debate about the usefulness of variation in sacral number, sternal fusion, and fibulo-calcaneal contact in oviraptorid taxonomy[27]. In all of these features, it contrasts other specimens of *Heyuannia yanshini* in that it has fewer sacral vertebrae (6 vs. 8), unfused sterna, and no extensive fibulocalcaneal contact. Furthermore, it differs from *Heyuannia yanshini* in that the third metacarpal is shorter than the second metacarpal, and is reduced in width. All of these features are identical to the states in *Oksoko avarsan*, and its referral to this taxon reduces variation in *Heyuannia yanshini*.

MPC-D 102/11 shares all of the unique cranial features of MPC-D 102/110, including the apically thickened cranial crest formed of the nasals, frontals, and parietals; the nasal recesses in a depression; the upturned frontal process of the postorbital; and the expanded jugal (Fig. S13). The axis is posteriorly concave, and the anterior post-axial cervicals are anteriorly concave, with large epipophyses, as in the holotype. The anatomy of the pelvis is identical, including the accessory brevis ridge, which is preserved on the ilium fragment of MPC-D 110-C. The ischium is identical in shape, straightness, and elongation. The femora are identical, as are the distal hindlimbs, including the lack of extensive fibulocalcaneal contact and an enlarged, bulb-like lateral process on the fourth distal tarsal. These features clearly indicate that it is referable to *Oksoko avarsan*.

MPC-D 102/12 shares numerous postcranial features with both MPC-D 102/11 and MPC-D 102/110 (Fig. S13). The cervical vertebrae are procoelous and have large epipophyses. The ilium has an accessory brevis ridge, and the ischium is long and straight. The hindlimb shares the low trochanteric ridge of the femur, the lack of extensive fibulocalcaneal contact, and the enlarged bulb on the lateral side of the fourth distal tarsal. The morphologies of the caudal vertebrae and chevrons is identical to MPC-D 102/11, except in the fusion of the pygal vertebrae and the relative elongation of the chevrons. Revisiting the site in 2018 resulted in the recovery of the left manual ungual I-2, which is identical to those of MPC-D 102/110.

7. Provenance of the Poached Specimens

While the specific localities from which the poached specimens were collected may never been known, multiple lines of evidence demonstrate that MPC-D 102/110 and MPC-D 102/11 are from the Nemegt Formation. Oviraptorids are only known from three formations in Mongolia: the Baruungoyot, Djadokhta, and Nemegt Formations. Both the Baruungoyot and Djadokhta Formations are characterized by their distinctively oxidized red sandstones. The Nemegt Formation, in contrast, is typified by coarse, grey fluvial sandstones, although some redbeds and some aeolian sediments are known to occur in the formation. MPC-D 102/110 and MPC-D 102/11 are preserved in a coarse, grey sandstone matrix, which is more characteristic of the Nemegt Formation than any other oviraptorid-bearing strata. Geochemical analysis also supports this conclusion. MPC-D 102/110 was incorporated into the geochemical analysis of Fanti et al.[24], which used X-Ray Diffraction to geochemically fingerprint specimens from known localities. Their analysis revealed that trace elements of fossil material can be used to confidently determine which formation the specimen was collected. Using this dataset, they estimated provenance for nine poached specimens, including MPC-D 102/110 (named Oviraptorid sp. in their study), and identified it as hailing from the Nemegt Formation with 88% certainty. Furthermore, their analysis suggested that the specimen was from the Nemegt locality with 90% certainty, although they did not include the western Nemegt Basin sites Bügiin Tsav and Guriliin Tsav in their analysis. Regardless, these data strongly support the Nemegt Formation provenance of MPC-D 102/110.

These conclusions are also supported by the specimens of known provenance, MPC-D 100/33 and MPC-D 102/12. These specimens are from Bügiin Tsav and Guriliin Tsav, respectively, and confirm that *Oksoko* was from the Nemegt Formation. Provenance of the poached specimens could be confirmed by future relocation of the quarry.

8. Taphonomy of the Holotype

The taphonomic circumstances surrounding the type specimen cannot be directly assessed because the quarry from which it was poached is unknown. However, some details can be gleaned from the bones themselves. Numerous lines of evidence indicate that the specimens were buried rapidly without being transported. The surfaces of the bones show no evidence of weathering or abrasion, and and there is no evidence of modification by insects or scavengers, indicating minimal subaerial exposure. The bones show no preferred orientation and are completely and tightly articulated, although they have been slightly crushed and skewed post-depositionally. There are no biases in skeletal representation, as even the delicate sclerotic plates are preserved. The positions of the skeletons differ from those of other theropod bonebeds, where individuals are usually preserved in the death pose or are slightly disarticulated, and buried subsequent to death[28,29]. The *Sinornithomimus* bonebed in China preserves skeletons in life position, but these skeletons are mired, as indicated by hindlimbs plunging into the sediment[30,31]. In contrast, the hindlimbs of MPC-D 102/110 lie flat, parallel to the bedding plane, and were clearly resting on a hard surface, rather than being trapped in soft sediment. The cause of death cannot be directly ascertained, but the tight huddle of the specimens may point to exposure as a possible cause. Regardless, the cause of death must have been non-violent, because the specimens were resting when killed and subsequently buried.

9. Table of age ranges

Species	FAD	LAD	Reference
Herrerasaurus_ischigualastensis	235	228	32
Fukuivenator	127	115	33
Guanlong	163	157	34
Sinosauropteryx	124	122	35
Gallimimus	72.1	66	36
Haplocheirus	163	157	37
Jianchangosaurus	129	122	38
Alxasaurus	113	100	39
Velociraptor_mongoliensis	83.6	74	40
Incisivosaurus_gauthieri	126	113	41
Similicaudipteryx_yixianensis	126	113	42
Caudipteryx_zoui	126	113	43
Caudipteryx_dongi	126	113	44
Avimimus_portentosus	74	68	36
Avimimus_nemegtensis	72.1	66	36
Microvenator_celer	115	108	45
Gigantoraptor_erlianensis	86.3	83.6	46
Chirostenotes_pergracilis	76.6	74.8	47
Hagryphus_giganteus	76.6	74.8	48
Nomingia_gobiensis	72.1	66	36
Citipes_elegans	76.6	74.8	47
Elmisaurus_rarus	72.1	66	36
Apatoraptor_pennatus	74	73	49
Caenagnathasia_martinsoni	93.9	86.3	20
Epichirostenotes_curriei	73	72.1	49
Caenagnathus_collinsi	76.6	74.8	47
Anzu_wyliei	68	66	14
Nankangia_jiangxiensis	83.6	66	50
Oviraptor_philoceratops	83.6	74	40
Yulong_mini	83.6	66	51
Wulatelong_gobiensis	83.6	72.1	52
Rinchenia_mongoliensis	72.1	66	36
Tongtianlong_limosus	83.6	66	23
Ganzhousaurus_nankangensis	83.6	66	53
Citipati_osmolskae	83.6	74	54
Zamyn_Khondt_oviraptorid	83.6	74	40
Huanansaurus_ganzhouensis	83.6	66	55
Corythoraptor_jacobsi	83.6	66	2
Shixinggia_oblita	72.1	66	56
Khaan_mckennai	83.6	74	54
Conchoraptor_gracilis	74	72.1	36
Machairasaurus_leptonychus	83.6	74	57
Nemegtomaia_barsboldi	72.1	66	36
Heyuannia_huangi	72.1	66	58
Heyuannia_yanshini	74	72.1	36
Banji_long	83.6	66	59
Jiangxisaurus_ganzhouensis	83.6	66	60
Oksoko avarsan	72.1	66	36

10. Measurements of *Oksoko avarsan*

Table S1. Selected measurements (mm) of known specimens of *Oksoko avarsan*.

Specimen	MPC-D 102/110.a	MPC-D 102/110.b	MPC-D 102/11	MPC-D 102/12	MPC-D 100/33
Skull length	150	162			
Skull height	94	85			
Preorbital skull length	69	70.7			
Postorbital skull length	57	43	46		
Orbit length	47	38.6	45		
Mandible length	120				
Ceratobranchial length	55.91				
Atlas–Axis length					29.4
C3 centrum length				30.9	22.7
C4 centrum length				30.4	24.7
C5 centrum length				30.5	26.6
C6 centrum length				28.9	27.1
C7 centrum length				29.8	
C8 centrum length				28.1	
C9 centrum length				27.8	
C10 centrum length					
Dorsal series length				293.95	258.9
Sacrum length			146.9		162.2
Caudal series length			435est	527est	511est
Scapulacoracoid length					205
Furcula transverse width					98
Humerus length		113			128.7R/129.3L
Radius length	92	90			94.7
Ulna length	92	94			99
MC 1 length	23.5R/23.95L	24			23.8
MC 2 length	37.35R/40.09L	39.71			41.7
MC 3 length	23.73R/28.25L				32
I-1 length	30.1R/32.22L	31.26			27.7
I-2 length	31.7R/31.2L	35.2		62	39.5
II-1 length	20.96R/20.82L	20.6			17.5
II-2 length	17.8R/17.6L	15			14.5
II-3 length	17.7R/18.7L	18			20
III-1 length	9.07	9.23			
Ilium length			216	319	
Ilium height above acetabulum			61.9	89.6	
Pubis length	180+	170+	226		210
Ischium length	127+	169	166		177
Femur length	235	224	210	280	233
Femur circumference	86est	87est	78	104	82
Tibia length	275	270	259	315	267
Astragalus height	84	87est	77	87.6	73
Astragalus width	46	42.6	40.2	59.5	48
Metatarsal I length	26	30.2	20.3		29.2
Metatarsal II length	112	104	102	130	111.5
Metatarsal III length	127.5	118	117	146.4	124.5
Metatarsal IV length	119	110	112	134.2	114.5
Metatarsal V length	38	45			44
Pedal digit 1 length	38	39	31.4+		44.9
Pedal digit 2 length	85.4	79.3	80.8		92.8
Pedal digit 3 length	110.4	107.2	108.2		112.2
Pedal digit 4 length	83.2	82.6	80		84.3

11. Character List (from Osmólska et al. 2004; modifications are cited)

1. Ratio of the preorbital skull length to the basal skull length: 0.6 or more (0); less than 0.6 (1) (Lü et al., 2013 [50]).

2. Pneumatized crest-like prominence on the skull roof: absent (0); present (1).

3. Ratio of the width (across premaxilla–maxilla suture) of the snout to its length: less than 0.3 (0); 0.3–0.4 (1); more than 0.4 (2) (Lü et al., 2013). (ORDERED)

4. Ratio of the length of the tomial margin of the premaxilla to the premaxilla height (ventral to the external naris): 0.7 or less (0); 1.0–1.4 (1); more than 1.7 (2). (ORDERED)

5. Inclination of the anteroventral margin of the premaxilla relative to the horizontally positioned ventral margin of the jugal: vertical (0); posterodorsal (1); anterodorsal (2).

6. Ventral projection of the premaxilla below the ventral margin of the maxilla: absent (0); present (1).

7. Ventral projection of the premaxilla below the ventral margin of the maxilla: small (0); significant (1)

8. Share of the premaxilla (ventral) in the basal skull length: 0.10 or less (0); 0.12 or more (1).

9. Pneumatization of the premaxilla: absent (0); present (1).

10. Ratio of the length of the maxilla (in lateral view) to the basal skull length: 0.4–0.7 (0); less than 0.4 (1) (Lü et al., 2013).

11. Subantorbital portion of the maxilla: not inset medially (0); inset medially (1).

12. Palatal shelf of the maxilla with two longitudinal ridges and a tooth-like ventral process: absent (0); present (1).

13. Ventral margins of maxilla and jugal: margins form a straight line (0); the ventral margin of the maxilla slopes anteroventrally, its longitudinal axis at an angle of ca. 120°to the longitudinal axis of the jugal (1).

14. Rim around antorbital fossa: well pronounced (0); poorly delimited (1).

15. Antorbital fossa: bordered anteriorly by the maxilla (0); bordered anteriorly by the premaxilla (1).

16. Accessory maxillary fenestrae: absent (0); at least one accessory fenestra present (1).

17. Nasal along midline: longer than frontal (0); shorter than or as long as the frontal (1).

18. Nasals: separate (0); fused (1).

19. Subnarial process of the nasal: long (0); short (1).

20. Shape of the narial opening: longitudinally oval (0); teardrop-shaped, slightly longer than wide (1); much longer than wide (2). (ORDERED)

21. Nasal recesses: absent (0); present (1).

22. External naris position relative to the antorbital fossa: naris and fossa widely separated (0); posterior margin of the naris reaching the fossa (1); overlapping anterodorsally most of the fossa (2). (ORDERED)

23. Ventral margin of the external naris: at the level of the maxilla (0); dorsal to the maxilla (1).

24. Prefrontal: present (0); absent or fused with the lacrimal (1).

25. Lacrimal shaft: not projecting outward beyond the orbital plane and lateral surface of the snout (0); the middle part of the shaft projecting laterally to form a flattened transverse bar in front of the eye (1).

26. Lacrimal recesses: absent (0); present (1).

27. Ratio of the length of the orbit to the length of the antorbital fossa: 0.7–0.9 (0); 1.2 or more (1).

28. Ratio of the length of the parietal to the length of the frontal: 0.6 or less (0); 1.0 or more (1).

29. Pneumatization of skull roof bones: absent (0); present (1).

30. Sagittal crest along the interparietal contact: absent (0); present (1).

31. Supratemporal fossa: invading the frontal (0); not invading the frontal (1).

32. Infratemporal fenestra: dorsoventrally elongate, narrow anteroposteriorly (0); subquadrate, its anteroposterior length comparable to the orbital length (1).

33. Pneumatization of the squamosal: absent (0); present (1).

34. Cotyle-like incision on the ventrolateral margin of the squamosal (for reception of the dorsal end of the ascending process of the quadratojugal): absent (0); present (1).

35. Ventral ramus of the jugal: deep dorsoventrally and flattened mediolaterally (0); shallow dorsoventrally or rod-shaped (1).

36. Jugal process of the postorbital: not extending ventrally below two-thirds of the orbit height (0); long, extending ventrally close to the base of the postorbital process of the jugal (1).

37. Postorbital process of the jugal: posterodorsally inclined (0); perpendicular to the ventral ramus of the jugal (1).

38. Postorbital process of the jugal: present (0); absent (1).

39. Jugal–postorbital contact: present (0); absent (1).

40. Quadratojugal process of the jugal in lateral view: forked (0); not forked (1); fused with the quadratojugal (2).

41. Quadratojugal–squamosal contact: absent (0); present (1).

42. Ascending (squamosal) process of the quadratojugal: bordering ca. the ventral half, or less, of the infratemporal fenestra (0); bordering the ventral two-thirds or more of the infratemporal fenestra (1).

43. Ascending (squamosal) process of the quadratojugal: present (0); absent (1).

44. Angle between the ascending and jugal processes of the quadratojugal: ca. 90° (0); less than 90°(1).

45. Quadrate process of the quadratojugal: well developed, extending posteriorly or posteroventrally beyond the posterior margin of the ascending process (0); not extending beyond the posterior margin of the ascending process (1).

46. Dorsal part of the quadrate: erect (0); bent backward (1).

47. Otic process of the quadrate: articulating only with the squamosal (0); articulating with the squamosal and the lateral wall of the braincase (1).

48. Pneumatization of the quadrate: absent (0); present (1).

49. Lateral accessory process on the distal end of the quadrate for articulation with the quadratojugal: absent (0); present (1).

50. Lateral cotyle for the quadratojugal on the quadrate: absent (0); present (1).

51. Mandibular condyles of quadrate: posterior to the occipital condyle (0); in the same vertical plane as the occipital condyle (1); anterior to the occipital condyle (2). (ORDERED)

52. Nuchal transverse crest: pronounced (0); not pronounced (1).

53. Occiput position in relation to the ventral margin of the jugal–quadratojugal bar: about perpendicular (0); inclined anterodorsally (1).

54. Paroccipital process: directed laterally (0); directed ventrally (1).

55. Foramen magnum: smaller than or equal in size to the occipital condyle (0); larger than the occipital condyle (1).

56. Basal tubera: modestly pronounced (0); well developed, widely separated (1).

57. Pneumatization of the basisphenoid: weak or absent (0); extensive (1).

58. Basipterygoid processes: well developed (0); strongly reduced (1).

59. Basipterygoid processes: present (0); absent (1).

60. Parasphenoid rostrum: horizontal or anterodorsally directed (0); sloping anteroventrally (1).

61. Depression in the periotic region: absent (0); present (1).

62. Pneumatization of the periotic region: absent or weak (0); extensive (1).

63. Quadrate ramus of the pterygoid: distant from the braincase wall (0); overlapping the braincase (1).

64. Pterygoid basal process for contact with the basisphenoid: absent (0); present (1).

65. Ectopterygoid position: lateral to the pterygoid (0); anterior to the pterygoid (1).

66. Ectopterygoid contacts with the maxilla and lacrimal: absent (0); present (1).

67. Ectopterygoid: short anteroposteriorly with a hook-like jugal process (0); elongate, shaped like a Viking ship, without a hook-like process (1).

68. Massive pterygoid–ectopterygoid longitudinal bar: absent (0); present (1).

69. Palate extending below the cheek margin: absent (0); present (1).

70. Palatine: tetraradiate or trapezoidal (0); triradiate, without a jugal process (1); developed in horizontal, longitudinal, and transverse planes perpendicular to each other (2).

71. Pterygoid wing of the palatine: dorsal to the pterygoid (0); ventral to the pterygoid (1).

72. Maxillary process of the palatine: shorter than the vomeral process (0); longer than the vomeral process (1).

73. Vomer: distant from the parasphenoid rostrum (0); approaching or in contact with the parasphenoid rostrum (1).

74. Suborbital (ectopterygoid–palatine) fenestra: well developed (0); closed or reduced (1).

75. Jaw joint: distant from the midline of the skull (0); close to the skull midline (1).

76. Movable intramandibular joint: present (0); suppressed (1).

77. Mandibular symphysis: loose (0); tightly sutured (1); fused (2). (ORDERED)

78. Extended symphyseal shelf at the mandibular symphysis: absent (0); present (1).

79. Downturned symphyseal portion of the dentary: absent (0); present (1).

80. U-shaped mandibular symphysis: absent (0); present (1).

81. Ratio of the length of the retroarticular process to the total mandibular length: less than 0.05 or the process absent (0); ca. 0.10 (1).

82. Dentary: elongate (0); proportionately short and deep, with maximum depth of dentary between 25% and 50% of dentary length (with length measured from the tip of the jaw to the end of the posterodorsal process) (1); extremely short and deep, with maximum depth 50% or more of dentary length (2) (ORDERED) (Longrich et al., 2013 [61]).

83. Ratio of the height of the external mandibular fenestra to the length of the fenestra: 0.2–0.5 (0); 0.7–1.0 (1).

84. External mandibular fenestra: present (0); absent (1).

85. Ratio of the length of the external mandibular fenestra to total mandibular length: absent or not more than 0.10 (0); between 0.15 and 0.20 (1), greater than 0.25 (2). (ORDERED)

86. Process of the surangular dividing the external mandibular fenestra: absent (0); short and broad (1); elongate and spike-like (2) (ORDERED) (Longrich et al., 2010 [57]).

87. Coossification of the articular with the surangular: absent (0); present (1).

88. Mandibular rami in dorsal view: straight (0); laterally bowed at midlength (1).

89. Anterodorsal margin of dentary in lateral view: straight (0); concave (1); broadly concave (2) (ORDERED) (Longrich et al., 2013).

90. Posterior margin of the dentary: incised, producing two posterior processes (0); oblique (1).

91. Posterodorsal process of the dentary long and shallow: present (0); absent (1).

92. Posteroventral process of the dentary shallow and long, extending posteriorly at least to the posterior border of the external mandibular fenestra: absent (0); present (1).

93. Coronoid process: posteriorly positioned and vertically projected (0); anteriorly positioned, near the midpoint of the jaw, with a medially hooked apex (1) (Longrich et al., 2013).

94. Surangular foramen: present (0); absent (1).

95. Mandibular articular facet for the quadrate: comprising the surangular and the articular (0); formed exclusively of the articular (1).

96. Mandibular articular facet for the quadrate: with one or two cotyles (0); convex in lateral view, transversely wide (1).

97. Position of the quadrate articular surface relative to the level of the adjoining dorsal margin of the mandibular ramus: ventral (0); moderately elevated, quadrate articulation grades smoothly into remainder of mandible (1); highly elevated, anterior and posterior margins of quadrate articulation at nearly right angles to remainder of mandible (2) (ORDERED) (Lamanna et al. 2014 [14]).

98. Anterior part of the prearticular: deep, approaching the dorsal margin of the mandible (0); shallow, strap-like, not approaching the dorsal mandibular margin (1).

99. Splenial: subtriangular, approaching the dorsal mandibular margin (0); strap-like, shallow, not approaching the margin (1).

100. Mandibular adductor fossa: anteriorly delimited, occupying the posterior part of the mandible (0); large, anteriorly and dorsally extended, not delimited anteriorly (1).

101. Coronoid bone: well developed (0); strongly reduced (1).

102. Coronoid bone: present (0); absent (1).

103. Premaxillary teeth: present (0); absent (1).

104. Maxillary tooth row: extends at least to the level of the preorbital bar (0); does not reach the level of the preorbital bar (1); maxillary teeth absent (2). (ORDERED)

105. Dentary teeth: present (0); absent from tip of jaw but present posteriorly (1); absent

(2) (ORDERED) (Longrich et al., 2013).

106. Number of cervicals (excluding cervicodorsal): not more than 10 (0); more than 10 (1).

107. Anterior articular facets of the centra in the anterior postaxial cervicals: not inclined or only slightly inclined (0); strongly inclined posteroventrally, almost continuous with the ventral surfaces of the centra (1).

108. Centra of the anterior cervicals: not extending posteriorly beyond their respective neural arches (0); extending posteriorly beyond their respective neural arches (1).

109. Epipophyses on the postaxial cervicals: in the form of a low crest or rugosity (0); prong-shaped (1).

110. Shafts of cervical ribs: longer than their respective centra (0); not longer than their respective centra (1).

111. Lateral pneumatic fossae (‘pleurocoels’) on the dorsal centra: absent (0); present (1).

112. Ossified uncinate processes on the dorsal ribs: absent (0); present (1).

113. Number of vertebrae included in the synsacrum in adults: not more than 5 (0); 6 (1); 7–8 (2). (ORDERED)

114. Sacral spines in adults: unfused (0); fused (1).

115. Lateral pneumatic fossae on the sacral centra: absent (0); present (1).

116. Transition point on the caudals: absent (0); present (1).

117. Number of caudals with transverse processes: 15 or more (0); fewer than 15 (1).

118. Lateral pneumatic fossae on the caudal centra: absent (0); present at least in the anterior part of the tail (1).

119. Neural spines confined to: at least 23 anterior caudals (0); at most 16 anterior caudals (1).

120. Number of caudals: more than 35 (0); 30 or fewer (1).

121. Posterior caudal prezygapophyses: overlapping less than half of the centrum of the preceding vertebra (0); overlapping at least half of the centrum of the preceding vertebra (1).

122. Hypapophyses in the cervicodorsal vertebral region: absent (0); small (1); prominent

(2). (ORDERED)

123. Posterior hemal arches: deeper than long (0); longer than deep (1).

124. Ratio of the length of the scapula to the length of the humerus: 0.7 or less (0); 0.8-1.1 (1), 1.2 or more (2). (ORDERED)

125. Acromion: projecting dorsally (0); projecting anteriorly (1); everted laterally (2).

126. Posteroventral process of the coracoid: absent or short, not extending beyond the glenoid diameter (0); long, posteroventrally extending beyond the glenoid (1).

127. Orientation of the glenoid on the pectoral girdle: posteroventral (0); lateral (1).

128. Deltopectoral crest: low, its width equal to, or smaller than, the shaft diameter (0); expanded, wider than the shaft diameter (1).

129. Extent of the deltopectoral crest (measured from the humeral head to the apex of the crest): about the proximal third of the humerus length or less (0); ca. 40%–50% of the humerus length (1).

130. Shaft of the ulna: straight (0); bowed, convex posteriorly (1).

131. Ratio of the length of the radius to the length of the humerus: 0.80 or less (0); 0.85 or more (1).

132. Combined lengths of manual phalanges III-1 and III-2: greater than the length of phalanx III-3 (0); less than or equal to the length of phalanx III-3 (1).

133. Ratio of the length of metacarpal I to the length of metacarpal II: 0.5 or more (0); less than 0.5 (1).

134. Proximal margin of metacarpal I in dorsal view: straight, horizontal (0); angled due to a medial extent of the carpal trochlea (1).

135. Metacarpal II relative to metacarpal III: shorter (0); subequal (1); longer (2). (ORDERED)

136. Ratio of the length of metacarpal II to the length of the humerus: 0.4 or less (0); more than 0.4 (1).

137. Ratio of the length of the manus to the length of the humerus plus the radius: less than 0.50 (0), between 0.50 and 0.65 (1), greater than 0.65 (2). (ORDERED)

138. Ratio of the length of the manus to the length of the femur: 0.3–0.6 (0); more than 0.7 (1).

139. Ratio of the length of the humerus to the length of the femur: 0.50–0.69 (0); 0.70 or

more (1) (Lü et al., 2013).

140. Dorsal margins of opposite iliac blades: well separated from each other (0); close to or contacting each other along their medial sections (1).

141. Dorsal margin of the ilium along the central portion of the blade: straight (0); arched (1); concave (2) [modified based on Nomingia]

142. Preacetabular process of the ilium relative to the postacetabular process (lengths measured from the center of the acetabulum): shorter or equal (0); longer (1).

143. Preacetabular process: not expanded or weakly expanded ventrally below the level of the dorsal acetabular margin (0); expanded ventrally well below the level of the dorsal acetabular margin (1).

144. Morphology of the ventral margin of the preacetabular process: cuppedicus fossa absent, margin transversely narrow (0); cuppedicus fossa or a wide shelf present (1); margin flat, wide at least close to the pubic peduncle (2).

145. Anteroventral extension of the preacetabular process: absent (0); present (1).

146. Anteroventral extension of the preacetabular process: with rounded tip (0); hook-like (1).

147. Posterior end of the postacetabular process: truncated or broadly rounded (0); narrowed or acuminate (1).

148. Anteroposterior length of the pubic peduncle: about the same as that of the ischial peduncle (0); distinctly greater than that of the ischial peduncle (1).

149. Dorsoventral extension of the pubic peduncle: level with the ischial peduncle (0); deeper than the ischial peduncle (1).

150. Ratio of the length of the ilium to the length of the femur: 0.50–0.79 (0); 0.80 or more (1) (Lü et al., 2013).

151. Pelvis: propubic (0); mesopubic (1); opisthopubic (2). (ORDERED)

152. Pubic shaft: straight (0); concave anteriorly (1).

153. Pubic foot: anterior process absent or shorter than posterior process (0); two processes equally long (1); anterior process longer than posterior process (2). (ORDERED)

154. Posterior margin of the ischial shaft: straight or almost straight (0); distinctly concave (1).

155. Greater trochanter of the femur: weakly separated, or not separated, from the femoral head (0); distinctly separated from the femoral head (1).

156. Anterior and greater trochanters: separated (0); contacting (1).

157. Dorsal extremity of the anterior trochanter: well below the greater trochanter (0); about level with the greater trochanter (1).

158. Fourth trochanter: well developed (0); weakly developed or absent (1).

159. Adductor fossa and the associated anteromedial crest on the distal femur: weak or absent (0); well developed (1).

160. Distal projection of the fibular condyle of the femur beyond the tibial condyle: absent (0); present (1).

161. Ascending process of the astragalus: as tall as wide across the base (0); taller than wide (1).

162. Distal tarsals: not fused with the metatarsus (0); fused with the metatarsus (1).

163. Proximal coossification of metatarsals II–IV: absent (0); present (1).

164. Arctometatarsus: absent (0); present, but only proximalmost extreme of metatarsal III obscured from anterior view in articulated metatarsus (1); present, proximal ~half of metatarsal III obscured from anterior view in articulated metatarsus (2) (Lamanna et al., 2014). (ORDERED)

165. Length of metatarsal I constituting: more than 50% of metatarsal II length (0); less than 50% of metatarsal II length (1); metatarsal I absent (2). (ORDERED)

166. Ratio of the maximum length of the metatarsus to the length of the femur: less than 0.3 (0), between 0.4 and 0.6 (1), 0.7-0.8 (2). (ORDERED)

167. Crenulated tomial margin of the premaxilla: absent (0); present (1).

168. Frontals: flat or weakly arched, not strongly projecting above orbit in lateral view (0); strongly arched, projecting well above orbit in lateral view to contribute to nasal–frontal crest (1).

169. Exoccipital: short, weakly projecting (0); strongly projects ventrally beyond squamosal in lateral view, approaching ventral end of quadrate (1).

170. Dentary posterodorsal ramus: straight or weakly curved (0); strongly bowed dorsally (1).

171. Dentary symphyseal ventral process: absent (0); prominent process present on

posteroventral surface of symphysis (1).

172. Dentary anteroventral margin in lateral view: straight or weakly downturned (0); strongly downturned (1).

173. Lateral surface of dentary: smooth (0); bearing a deep fossa, sometimes with associated pneumatopore (1).

174. Angular: contributes extensively to the border of the external mandibular fenestra (0); largely excluded by surangular (1).

175. Surangular with an anteroposteriorly elongate flange on the ventral edge: absent (0); present (1).

176. External mandibular fenestra: elongate (0); height subequal to length (1).

177. Dentary contribution to external mandibular fenestra relative to length of dentary: no more than 50% (0); exceeds 50% (1).

178. Metacarpal I expanded ventrally to cover ventral surface of metacarpal II: absent (0); present (1).

179. Unguals of manual digits II and III: strongly curved (0); weakly curved (1).

180. Manual phalanx I-1: slender (0); more robust than II-1 (1); more than 200% diameter of II-1 (2) (ORDERED).

181. Manual phalanx III-3: longer than phalanx III-2 (0); shorter than or equal in length to III-2 (1).

182. Manual phalanx II-2: longer than II-1 (0); subequal to or slightly shorter than II-1 (1); distinctly shorter than II-1 (2) (ORDERED) (Longrich et al., 2013).

183. Manual digit II: elongate, with combined lengths of manual phalanges II-1 and II-2 longer than metacarpal II (0); combined lengths of manual phalanges II-1 and II-2 subequal to metacarpal II (1) (Lamanna et al., 2014).

184. Ischium strongly bent posteriorly at midshaft, distal end forms an angle of at least 60owith proximal end: absent (0); present (1).

185. Metatarsus: elongate (0); short, length does not exceed 300% of proximal width (1).

186. Ilium: tall (0); low and anteroposteriorly elongate, height less than 25% of length (1).

187. Anterior blade of ilium shallower than posterior blade: absent (0); present (1).

188. External naris: placed anteriorly (0); extends posteriorly, with posterior end lying above antorbital fenestra (1).

189. Premaxillae, nasal processes anteroposteriorly expanded and mediolaterally compressed to form a bladelike internarial bar: absent (0); present (1).

190. Dentary, anterodorsal tip of beak: projecting upwards (0); projecting anterodorsally, tip of beak projecting at an angle of 45°or less relative to the ventral margin of the symphysis (1).

191. Dentary symphysis with interior surface bearing vascular grooves and associated foramina: absent (0); present (1).

192. Dentary symphysis bearing an hourglass-shaped ventral depression: absent (0); present (1).

193. Meckelian groove terminates: on the inside of the dentary (0); on the ventral surface of the symphysis (1).

194. Lingual triturating shelf: absent (0); present (1).

195. Symphyseal ridges inside the tip of the beak: absent (0); present but weakly developed (1); present and well developed (2) (ORDERED).

196. Lingual ridges inside the lateral occlusal surface of beak: absent (0); present (1).

197. Posteroventral process of dentary: straight (0); bowed ventrally (1).

198. Dentaries pneumatized: absent (0); present (1).

199. Dentary: participates in dorsal border of the external mandibular fenestra (0); excluded by anterior extension of the surangular (1).

200. Dentary: participates in ventral border of external mandibular fenestra (0); excluded by anterior extension of the angular (1).

201. Surangular and angular divided by posterior extension of the external mandibular fenestra: absent (0); present (1).

202. Posterior end of the surangular: deep (0); shallow, subequal to or shallower than angular (1).

203. Surangular: deep anteriorly (0); strap-like (1).

204. Retroarticular process extends: posteriorly (0); posteroventrally (1).

205. Metacarpal I: proportionately broad (0); long and slender, diameter 20% of length

(1).

206. Manual phalanx I-1: longer than II-2 (0); subequal to II-2 (1); shorter than II-2 (2) (ORDERED).

207. Ischiadic peduncle of pubis with prominent medial fossa: absent (0); present (?).

208. Ischium, obturator process located: distally (0); at midshaft of ischium (1).

209. Anterior margin of obturator process: straight or convex (0); distinctly concave (1).

210. Accessory trochanter of femur: weakly developed (0); prominent, subrectangular flange or finger-like process (1).

211. Metatarsal III: with an ovoid or subtriangular cross section (0); anteroposteriorly flattened, with a concave posterior surface (1).

212. Paroccipital process: elongate and slender, with dorsal and ventral edges nearly parallel (0); short and deep with convex distal end (1).

213. Mandibular articulation surface: as long as ventral end of quadrate (0); twice or more as long as quadrate surface, allowing anteroposterior movement of mandible (1).

214. Sternum, distinct lateral xiphoid process posterior to costal margin: absent (0); present (1)

215. Anterior edge of sternum: grooved for reception of coracoids (0); without grooves (1)

216. Deltopectoral crest: large and distinct, proximal end of humerus quadrangular in anterior view (0); less pronounced, forming an arc rather than being quadrangular (1).

217. Ischium: more than two-thirds of pubis length (0); two-thirds or less of pubis length (1)

218. Lateral ridge of femur: absent or represented only by faint rugosity (0); distinctly raised from shaft, mound-like (1)

219. Surangular, distinct groove on dorsal surface: present (0); absent (1).

220. Vomer, position: level with other palatal elements (0); ventral to other palatal elements (1)

221. Calcaneum: excludes astragalus from reaching lateral margin of tarsus (0); small process of astragalus protrudes through a circular opening in edge of calcaneum to reach lateral margin of tarsus (1).

222. Depression on lateral surface of dentary immediately anterior to external mandibular fenestra: absent (0); present (1).

223. Groove on ventrolateral edge of angular to receive posteroventral branch of dentary: absent (0); present (1).

224. Posteroventral branch of dentary twisted so that lateral surface of branch faces somewhat ventrally: absent (0); present (1).

225. Premaxilla, large, presumably pneumatic foramen at anteroventral corner of narial fossa: absent (0); present (1).

226. Accessory opening at anterodorsal extreme of snout: absent (0); present (1).

227. Development of symphyseal shelf of mandible: limited, anteroposterior length of mandibular symphysis (as measured on midline) less than 20% total anteroposterior length of mandible (0); intermediate, length of symphysis greater than 20% but less than 25% length of mandible (1); extensive, length of symphysis greater than 25% mandibular length (2) (ORDERED).

228. Prominent flange or shelf arising from lateral surface of dentary: absent (0); present (1).

229. Base of retroarticular process: considerably wider mediolaterally than tall dorsoventrally (0); approximately as wide as tall (1); considerably taller than wide (2). (ORDERED)

230. Posteriormost caudal vertebrae fused, forming a pygostyle-like structure: absent (0); present (1).

231. Humeral shaft: straight or nearly straight (0); strongly bowed laterally (1).

232. Proximodorsal extensor ‘lip’ on manual unguals: weak (i.e., continuous or nearly continuous with remainder of dorsal surface of ungual) and/or absent (0); prominent (‘set off’ from remainder of dorsal surface by distinct change in slope immediately distal to ‘lip’) (1).

233. Pubic process of ischium, ‘hooked’ anterodistal extension: absent (0); present (1).

234. Distal margin of obturator process: straight (0); distinctly concave, apex of obturator process angled distally (1).

235. Proximolateral edge of metatarsal IV attenuated into pointed process: absent (0); present (1).

236. Frontal anteriorly divided by slot for nasal and possibly lacrimal: absent (0); present (1).

237. Infradiapophyseal infraprezygapophyseal and infrapostzygapophyseal fossae on cervical and dorsocervical vertebrae: one or more absent (0); all three present (1).

238. Ratio of the length of the metatarsus to the length of the tibia: <0.5 (0); >0.5 (1).

239. Tibia ratio of the transverse width of the distal condyles to the length: 0.20 or greater (0); <0.20 (1).

240. Ratio of minimum transverse width to length of tarsometatarsus: >0.20 (0); <0.20 (1)

241. Fusion of distal tarsals III and IV at maturity: absent (0); present (1).

242. 'Hook-like' posterodorsal process of distal tarsal IV: absent (0); present (1).

243. Posterior protuberance on proximal end of tarsometatarsus caused by coossification of distal tarsals III and IV, plus MT II, III and IV: absent (0); present (1).

244. Anterior margin of metatarsal V in lateral view: straight or slightly curved (0); tightly curved (1).

245. Concavity on posterior surface of tarsometatarsus in cross section: absent or shallow (0); prominent and deep (1).

246. Transverse groove between flexor tubercle and proximal articular surface of manual ungual I-2: absent (0); present (1)

12. Character States of *Oksoko avarsan*

11?2111011 1110010100 1211111111 0011000001 1100011101 2001110101
111111111? ??00012011 1210221010 1111111??0 ??12211111 1112?10010 1010??1?10
0001120200 0001210000 1012111110 1110010111 1010101111
2-11011011 000?000000 0101100000 0001?00000 1?00100000 0010110011
0000000000 0000000

13. Supplementary References

Automatic citation updates are disabled. To see the bibliography, click Refresh in the Zotero tab.

14. Supplementary Figures

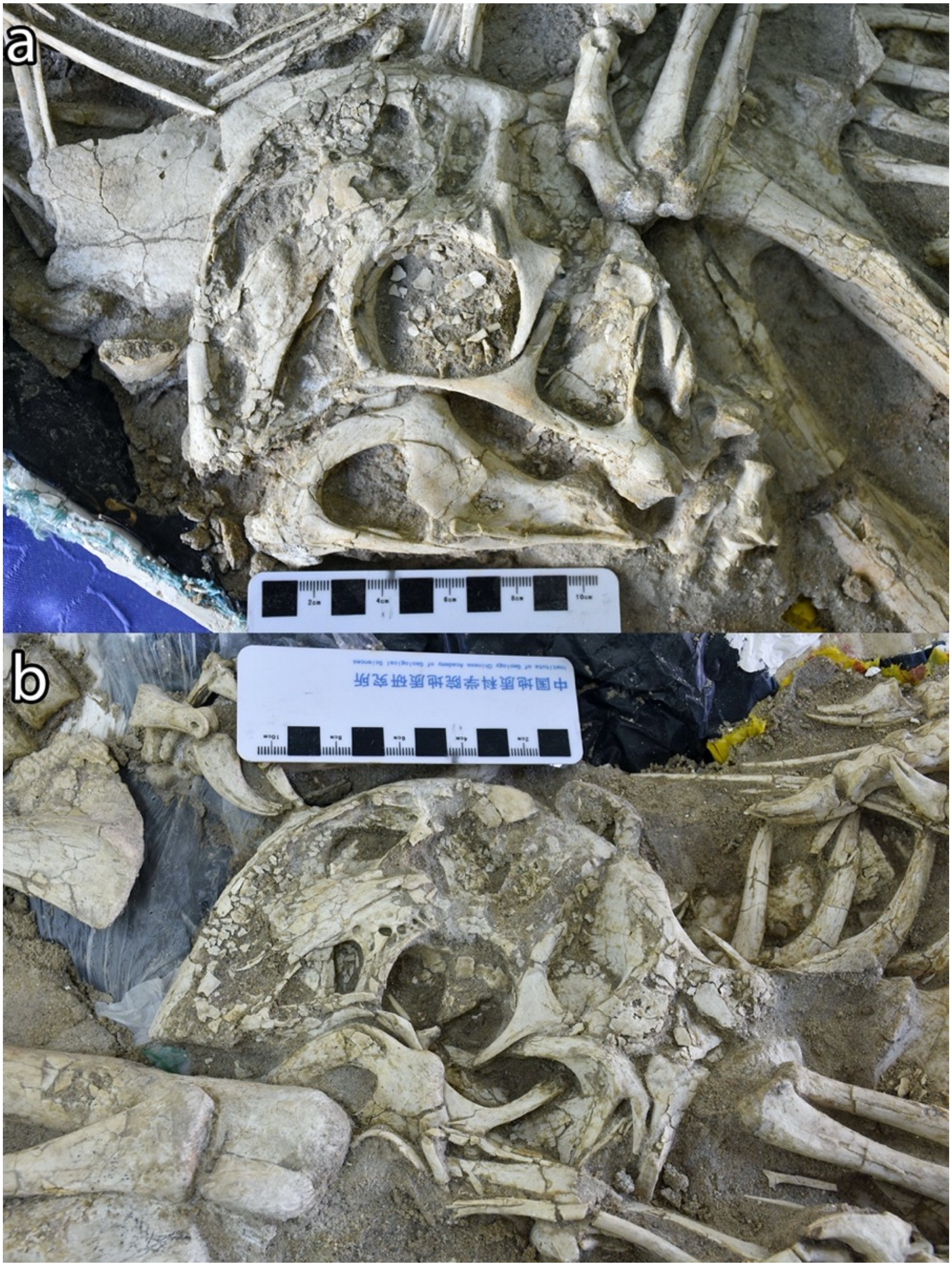

Fig. S1 | The skull of *Oksoko avarsan*. a, Skull of MPC-D 102/110.a (holotype) in left lateral view. **b,** Skull of MPC-D 102/110.b in left lateral view.

952

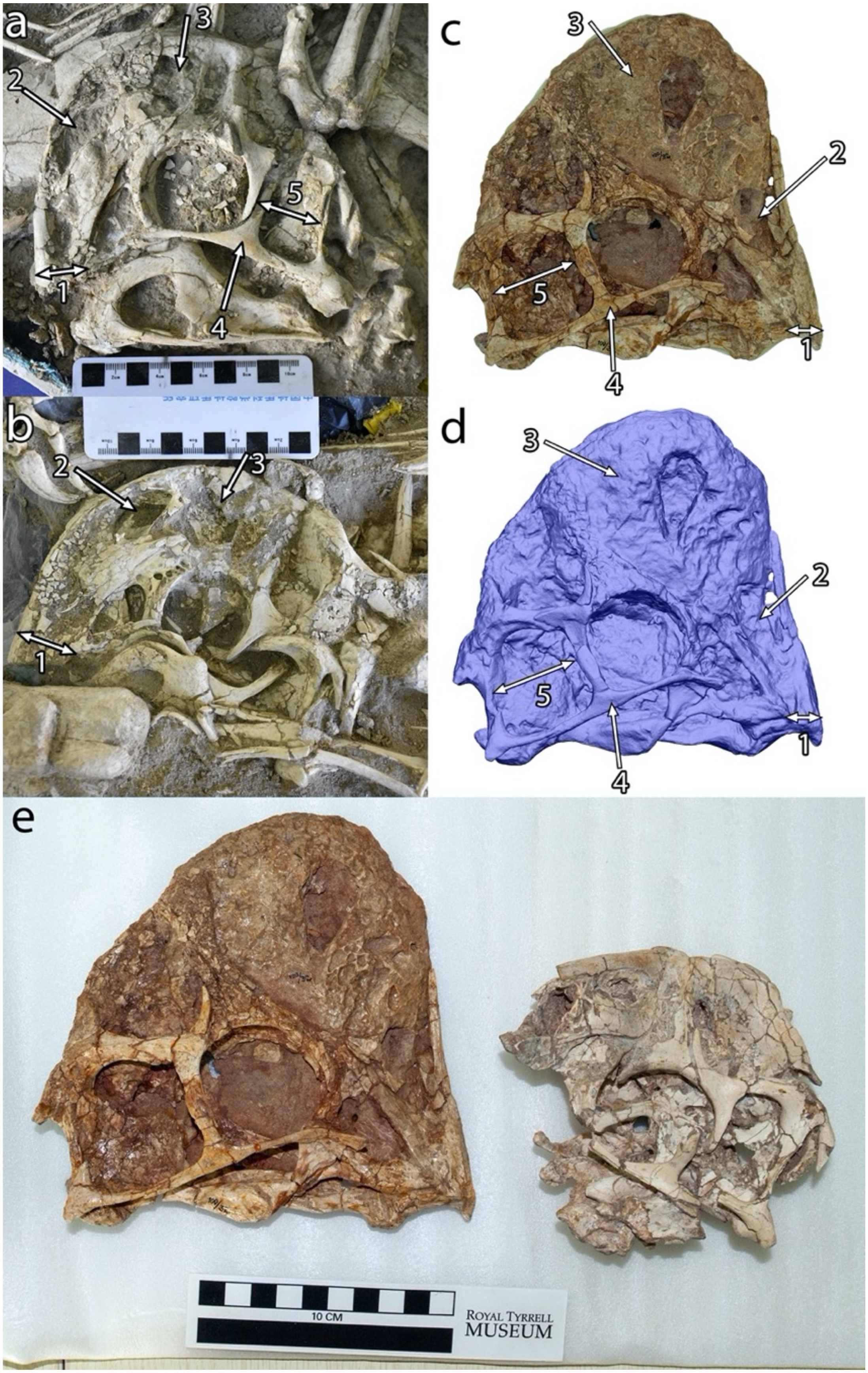

(Previous page) Fig. S2 | Comparison of the skulls of *Oksoko avarsan* and *Rinchenia mongoliensis*. a–d, skulls of *Oksoko avarsan* (**a, b**), and photogrammetric model of the skull of *Rinchenia mongoliensis* (**c, d**) in lateral view, showing five major features that distinguish the two taxa: 1) length of premaxilla at tomial edge; 2) position of naris; 3) size of lateral descending processes of the nasals; 4) expansion of the body of the jugal and presence of an interfingering contact with the quadratojugal; 5) size of infratemporal fenestra compared to skull and orbit length. **e,** comparison of skulls of MPC-D 100/32-a (*Rinchenia mongoliensis*; left) and MPC-D 102/11 (*Oksoko avarsan*; right), showing variation in crest size.

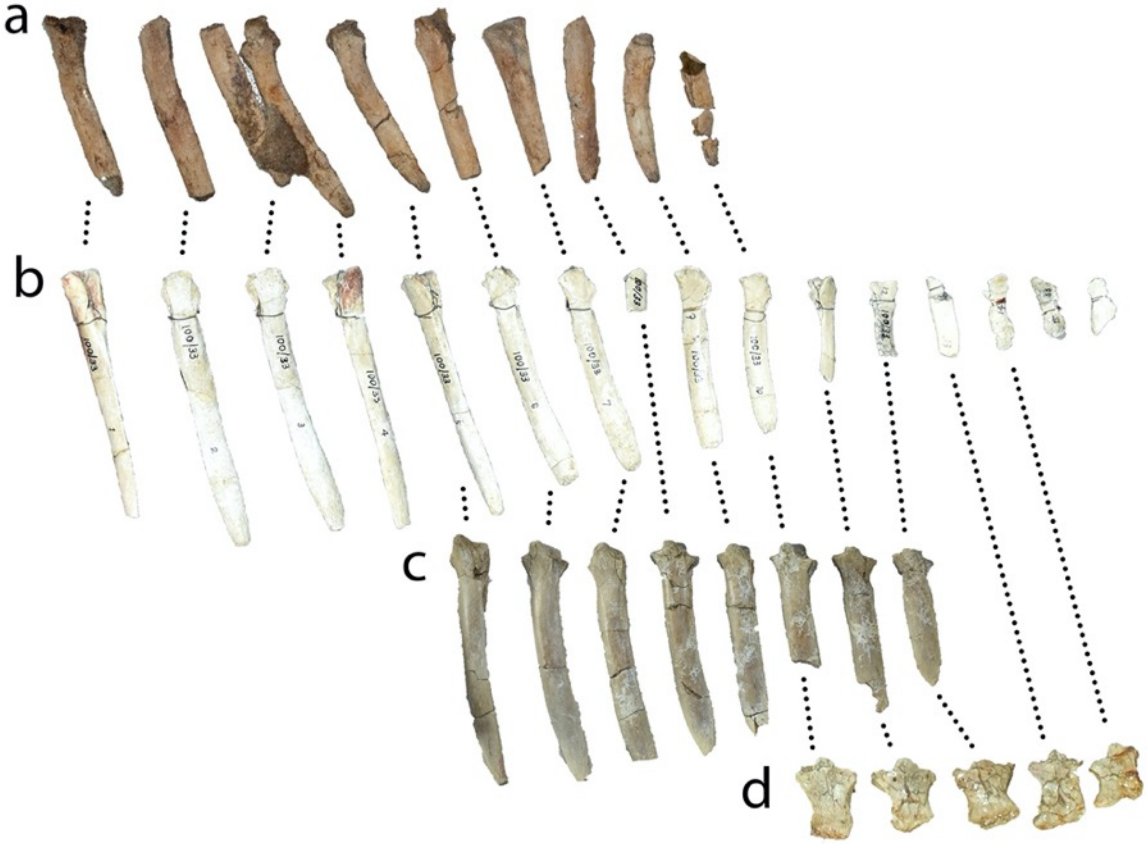

Fig. S3 | Comparison of the chevrons of *Oksoko avarsan* (a–c) and *Rinchenia mongoliensis* (d). Chevrons in left lateral view, showing elongate morphology in *Oksoko avarsan* (**a–c**) and platelike morphology in *Rinchenia mongoliensis* (**d**). Corresponding chevrons are connected by dotted lines. Note conserved morphology through ontogeny in *Oksoko avarsan* (**a–c**). Images not to scale.

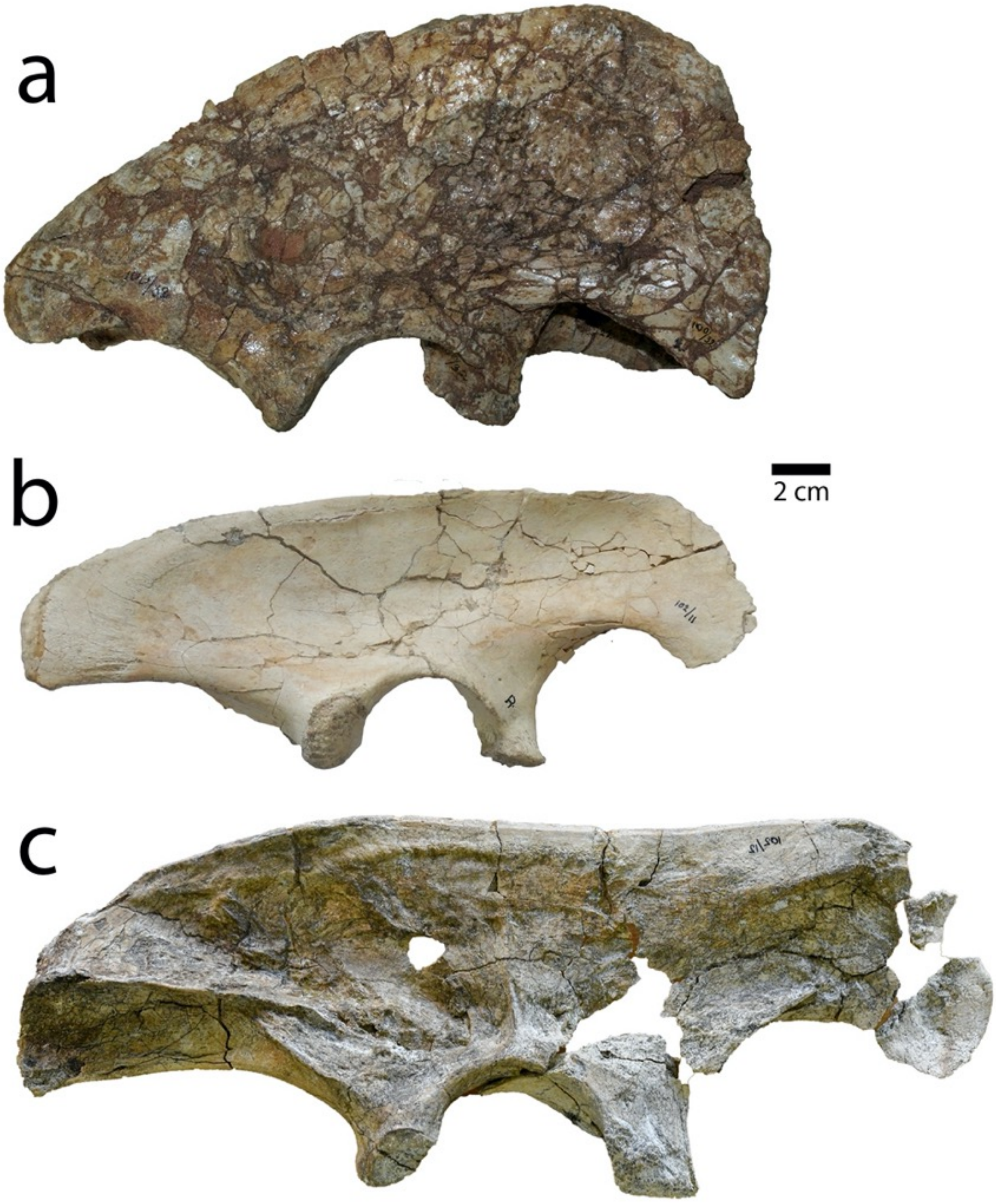

Fig. S4 | Comparison of the ilia of *Oksoko avarsan* (b, c) and *Rinchenia mongoliensis* (a). Ilia of MPC-D 100/32-a (*Rinchenia mongoliensis*, **a**), MPC-D 102/11 (*Oksoko avarsan*, **b**), and MPC-D 102/12 (*Oksoko avarsan*, **c**) in lateral (**a, b**) and medial (**c**) views**.** Note considerable variation in height between *Rinchenia mongoliensis* and *Oksoko avarsan*, despite similarity in size of MPC-D 100/32-a and MPC-D 102/11, and consistency in size between the juvenile MPC-D 102/11 (**b**) and the adult MPC-D 102/12 (**c**).

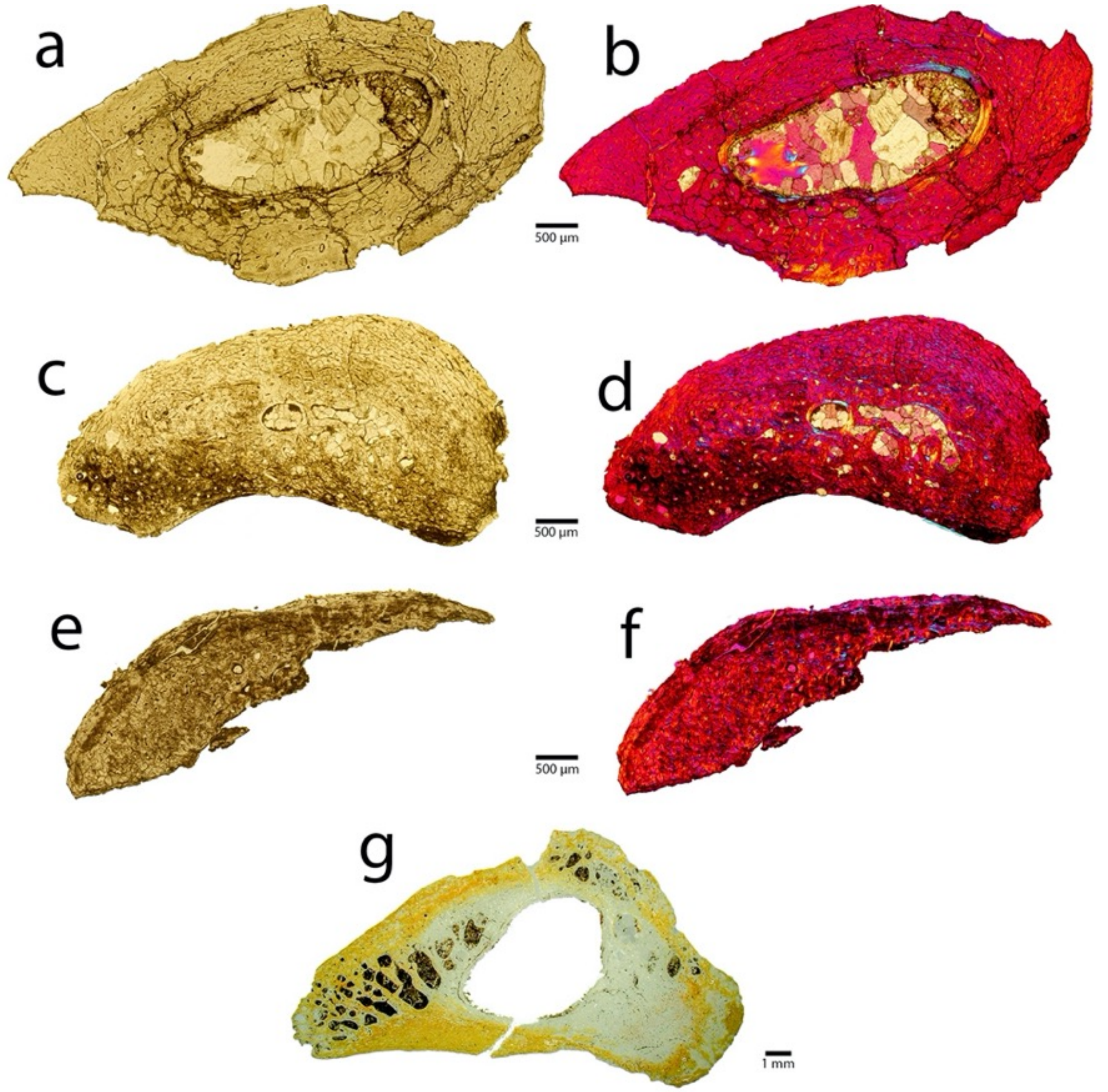

Fig. S5 | Overview of histological thin sections of the fibulae of *Oksoko avarsan*. Transverse thin sections of the fibulae of MPC-D 102/11 (**a, b**), MPC-D 102/110.a (**c, d**), MPC-D 102/110.b (**e, f**), and MPC-D 102/12 (**g**) under normal (**a, c, e, g**) and cross-polarized light with a lambda filter (**b, d, f**).

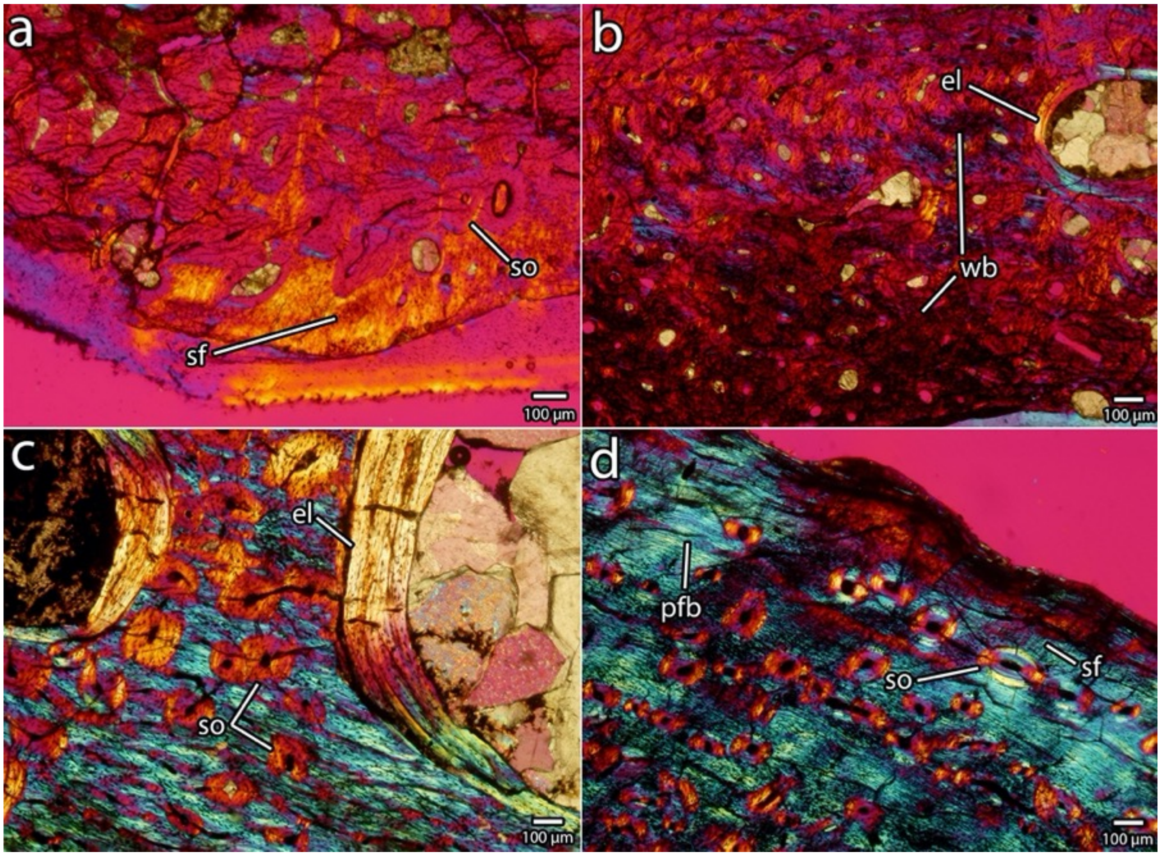

Fig. S6 | Histological details of *Oksoko avarsan*. a, Secondary osteons (**so**) and Sharpey's fibers (**sf**) in the periosteal cortex of the fibula of MPC-D 102/11. **b,** Woven bone (**wb**) and endosteal lamellae (**el**) in the inner cortex of the fibula of MPC-D 102/110.a. **c**, Secondary osteons (**so**) and endosteal lamellae (**el**) in the inner cortex of the fibula of MPC-D 102/12. **d**, Zone of secondary remodeling with secondary osteons (**so**), Sharpey's fibers (**sf**) and primary parallel-fibred bone at the periosteal surface of the femur of MPC-D 102/12. All images under cross-polarized light with a lambda filter.

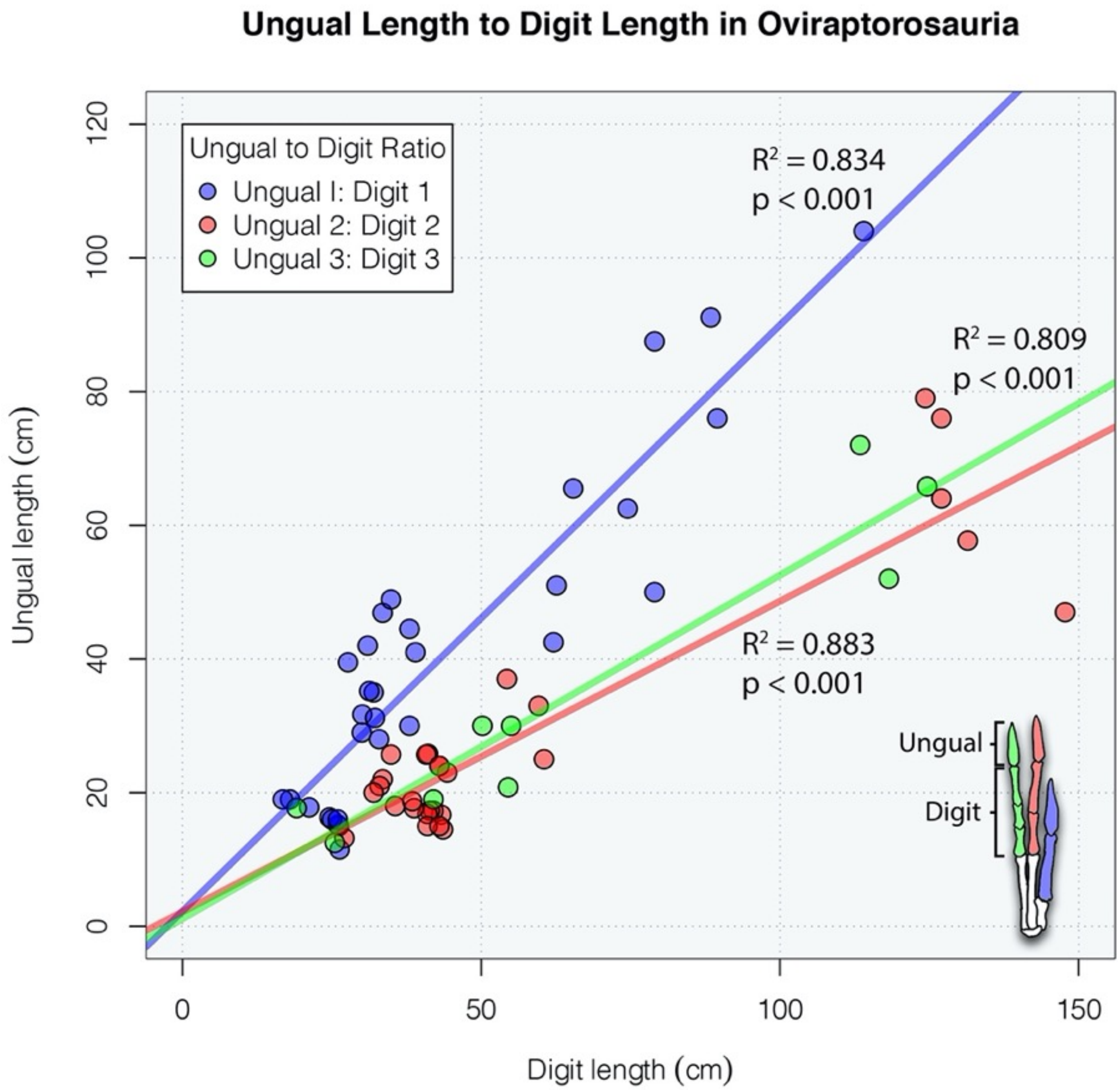

Fig. S7 | Bivariate plot of ungual length to length of the preceding digit. Straight-line measurement of the length of the ungual (y-axis) plotted against the sum of the lengths of non-ungual phalanges. R^2 values and significance are indicated for each line of best fit.

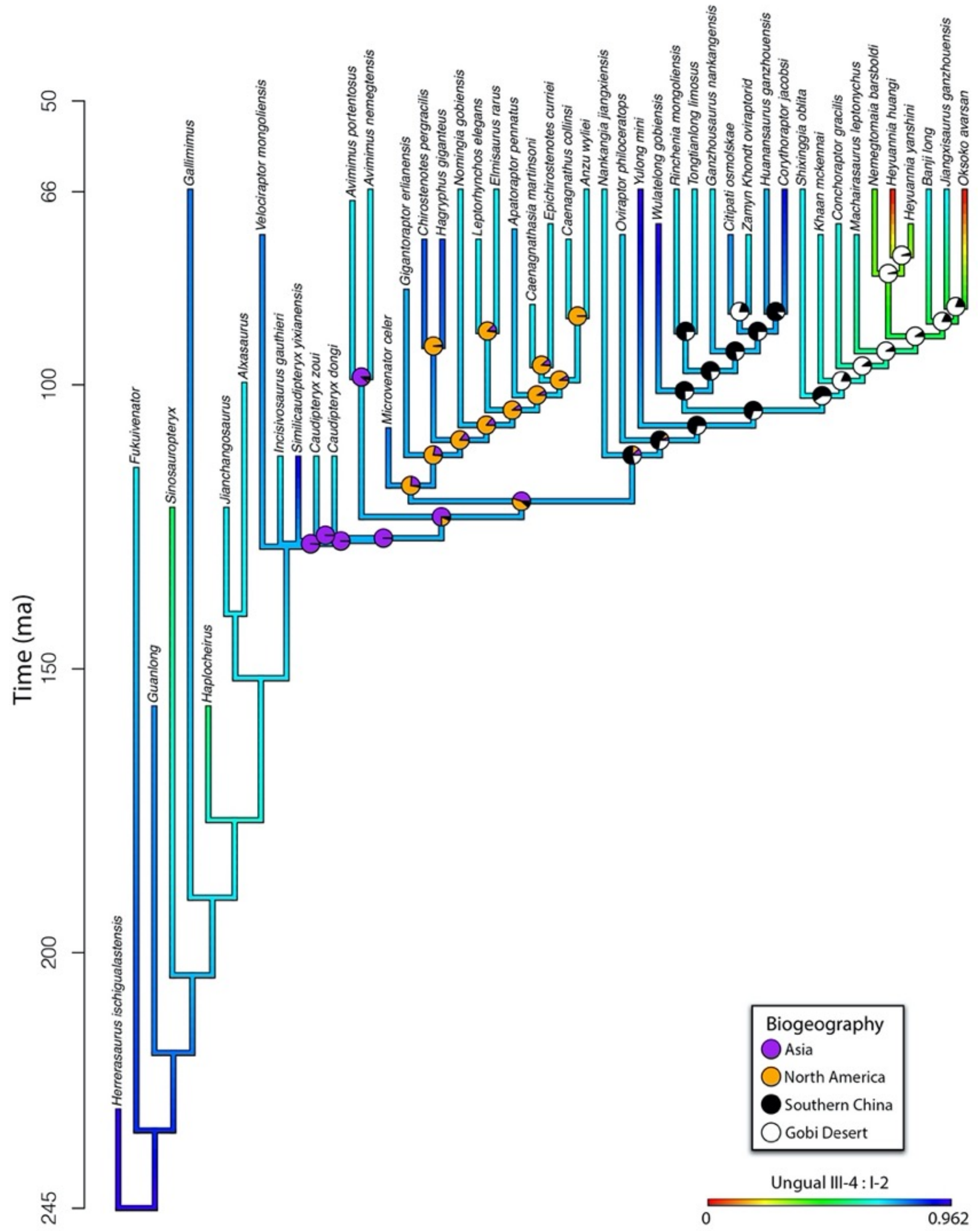

Fig. S8 | Ancestral state estimation of the ratio of manual ungual III-4 to manual ungual I-2. Complete time-calibrated tree, showing grafted outgroups (*Herrerasaurus* to *Alxasaurus*), and biogeographic estimates (pie charts). Branch colours show estimated ratio of manual ungual III-4 to I-2, with warm colours indicating a lower ratio (i.e. smaller digit III). Limits of the colour range are restricted to the range of conditions present within Oviraptorosauria.

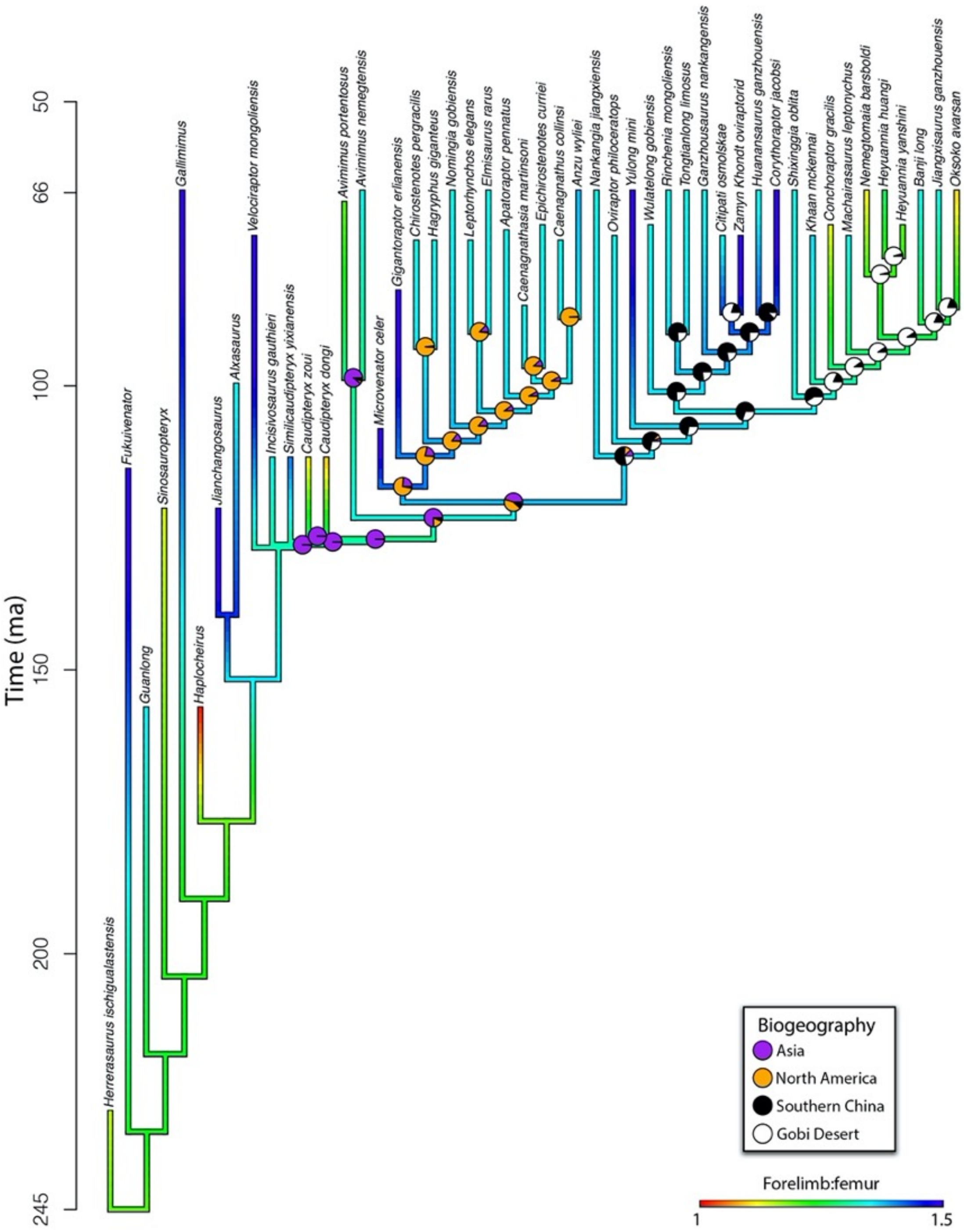

Fig. S9 | Ancestral state estimation of the ratio of forelimb length (humerus + ulna + metacarpal II) to femur length. Complete time-calibrated tree, showing grafted outgroups (*Herrerasaurus* to *Alxasaurus*), and biogeographic estimates (pie charts). Branch colours show estimated ratio of forelimb length to femur length, with warm colours indicating a lower ratio (i.e. shorter forelimb). Limits of the colour range are restricted to emphasize the range of conditions present within Oviraptorosauria—values falling into the green range are difficult to differentiate).

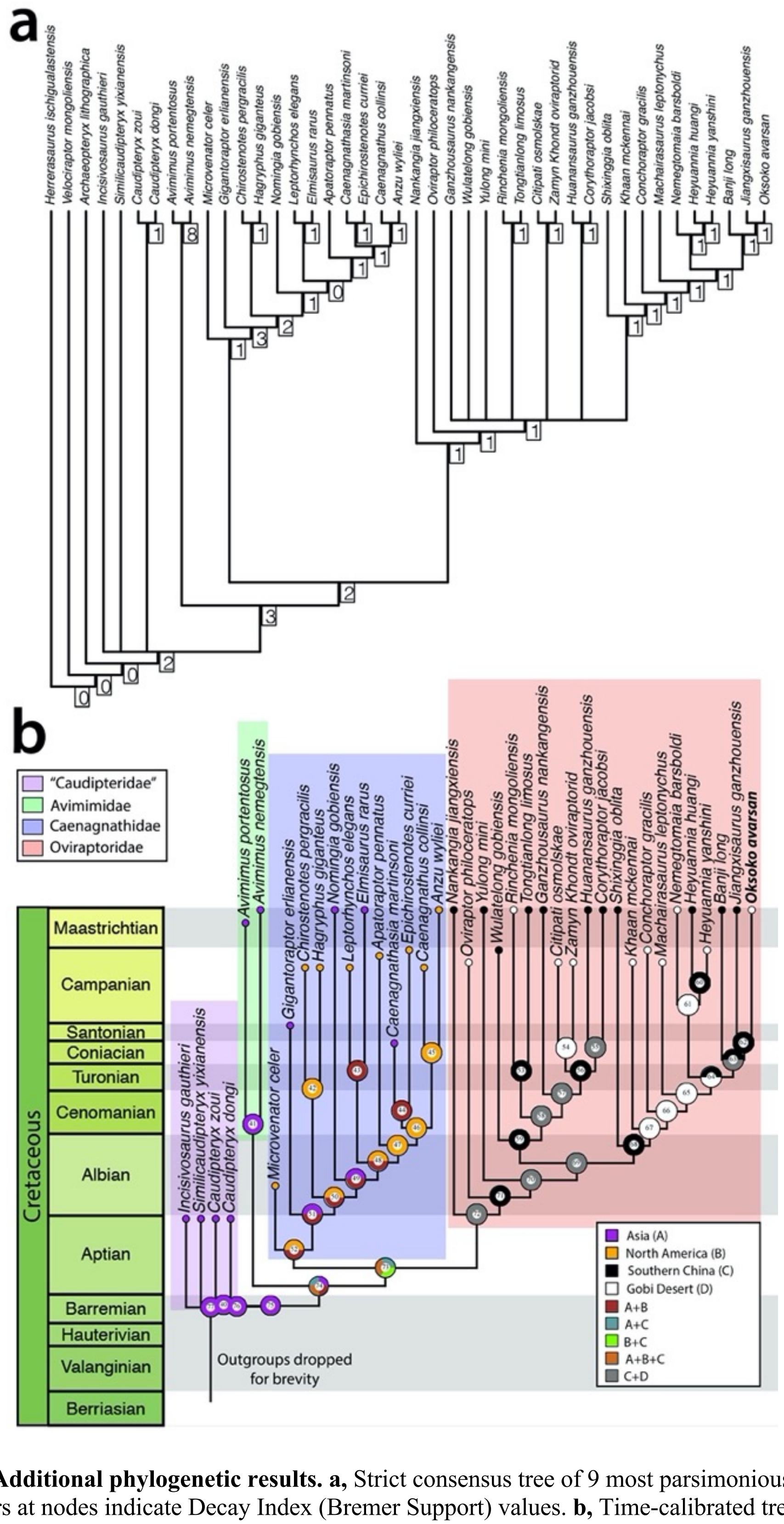

Figure S10 | Additional phylogenetic results. a, Strict consensus tree of 9 most parsimonious trees of 641 steps. Numbers at nodes indicate Decay Index (Bremer Support) values. **b,** Time-calibrated tree of Oviraptorosauria (with outgroups dropped), showing biogeographic reconstructions based on S-DIVA analysis, with tips showing known biogeographical provenance.

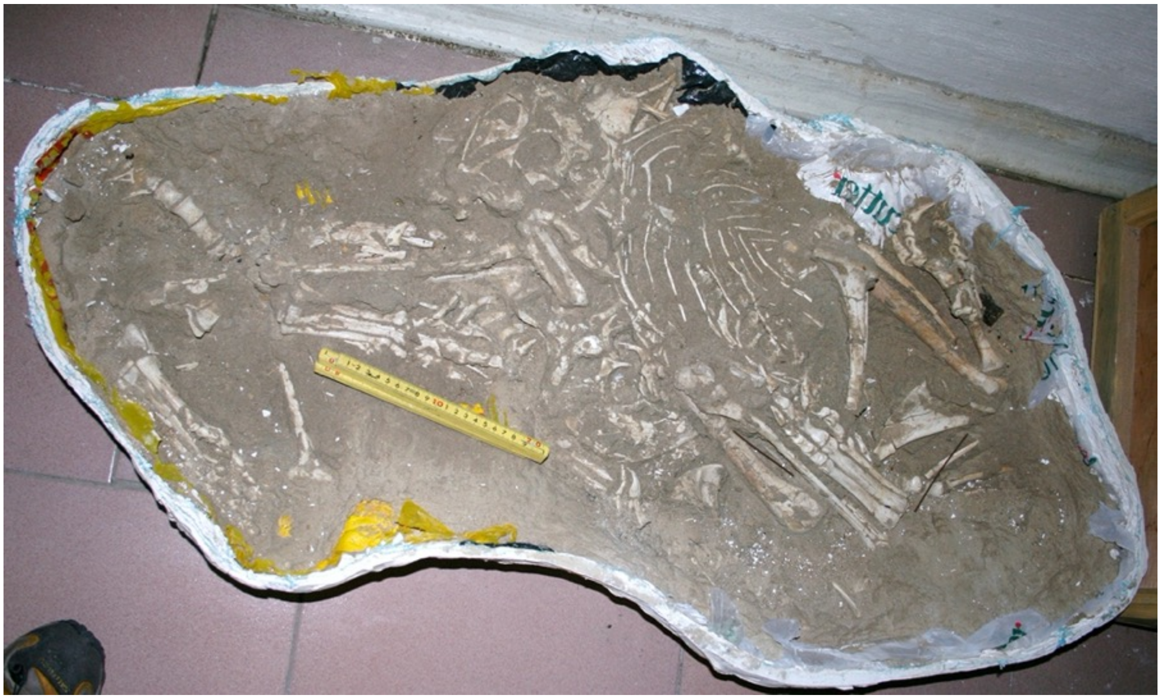

Fig. S11 | Holotype block (MPC-D 102/110) prior to final preparation. Note homogeneity of the sediment and field jacket, indicating single provenance and field-up direction of the block (exposed surface would have been down in the field). Scale bar is in centimeters.

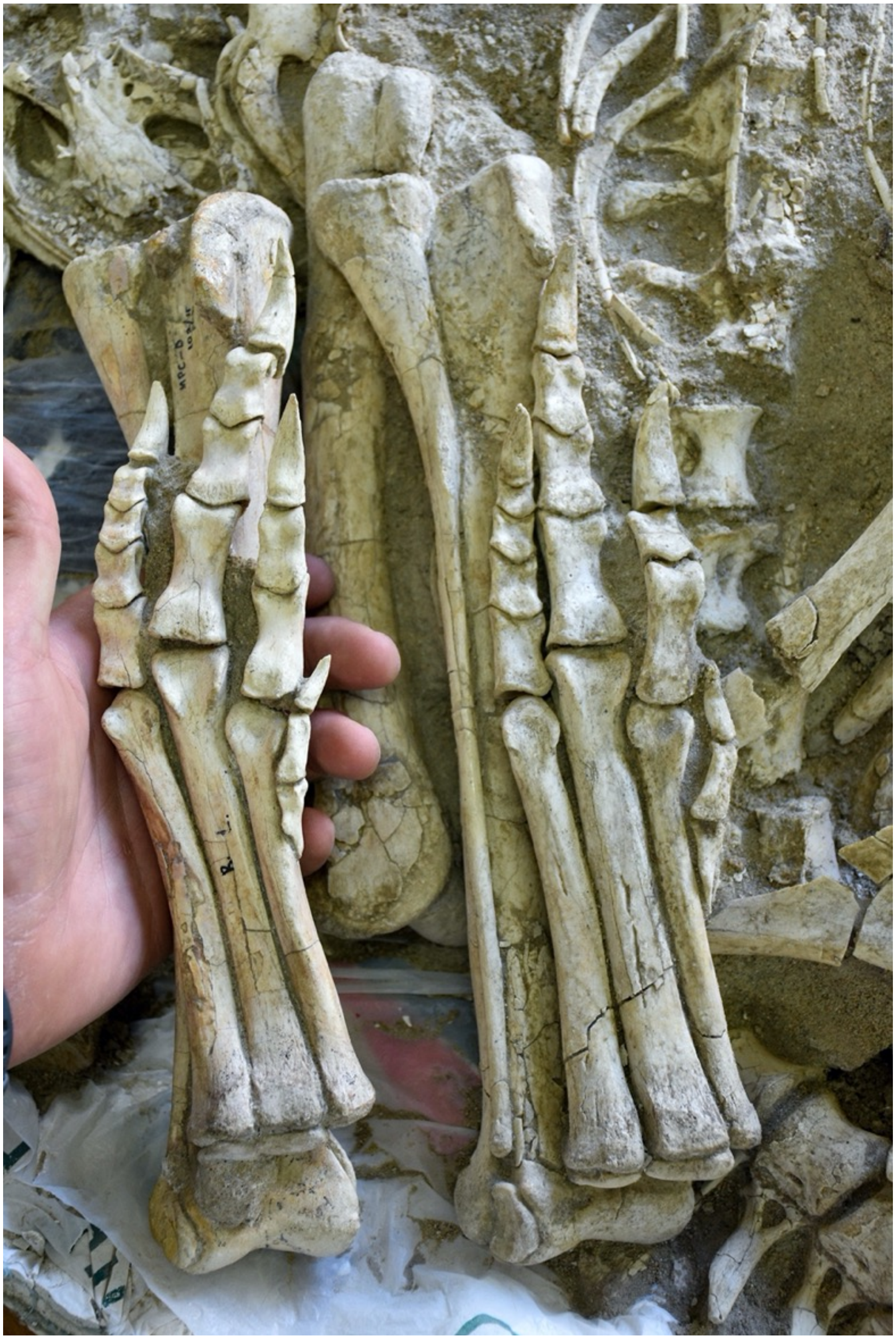

Fig. S12 | Conservation of pose in the holotype assemblage (MPC-D 102/110 + MPC-D 102/11). Right hindlimbs of MPC-D 102/11 (left) and MPC-D 102/110.a (right) showing highly conserved pose and crouched posture indicating minimal transport or taphonomic modification prior to burial. Note position of pedal digit III medial to cnemial crest, and identical preservation of the two specimens.

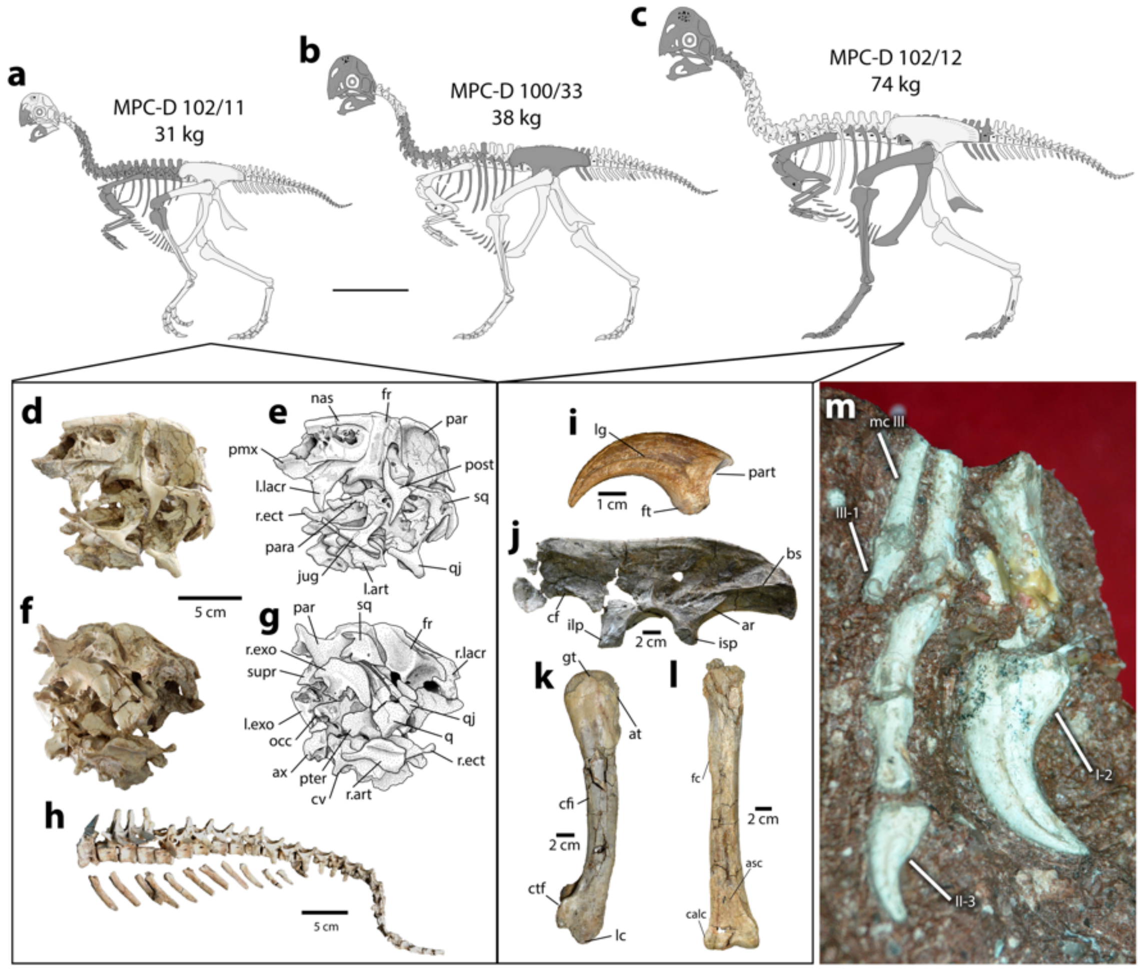

Figure S.13. Other known specimens of *Oksoko avarsan* and the manus of *Heyuannia huangi*. a–c, Skeletal reconstructions of MPC-D 102/11 (**a**), MPC-D 100/33 (**b**) and MPC-D 102/12 (**c**), showing variation in body mass and size. Missing elements are shaded. Scale bar is 25 cm. **d–g** Photographs (**d,f)** and interpretive illustrations (**e,g**) of the skull of MPC-D 102/11 in left lateral (**d,e**) and right posterolateral (**f,g**) views. **h,** Caudal series of MPC-D 102/11 in left lateral view. **i,** Manual ungual I-2 of MPC-D 102/12 in lateral view. **j,** Right ilium of MPC-D 102/12 in medial view, showing accessory brevis ridge. **k,** Right femur of MPC-D 102/12 in lateral view. **l,** Right tibia and astragalocalcaneum of MPC-D 102/12 in anterior view. **m,** Right manus of *Heyuannia huangi* (HYMV1-3) in medial view, showing presence of only a single phalanx on digit III. **Abbreviations: ar**, accessory ridge; **asc**, ascending process of astragalus; **at**, anterior trochanter; **ax**, axis; **bs**, brevis shelf; **calc**, calcaneum; **cf**, cuppedicus fossa; **cfi**, m. caudofemoralis insertion; **ctf**, crista tibiofibularis; **cv**, cervical vertebra; **fc**, fibular crest; **fr**, frontal; **ft**, flexor tubercle; **gt**, greater trochanter; **I-2**, manual ungual I-2; **II-3**, manual ungual II-3; **III-1**, manual phalanx III-1; **ilp**, iliac peduncle; **isp**, ishiadic peduncle; **jug**, jugal; **l.art**, left articular; **l.exo**, left exoccipital; **l.lacr**, left lacrimal; **lc,** lateral condyle; **lg**, lateral groove; **lt**, lateral trabecula; **mc III**, metacarpal III; **nas**, nasal; **occ**, occipital condyle; **par**, parietal; **para**, parabasisphenoid; **part**, proximal articulation; **pmx**, premaxilla; **post**, postorbital; **pter**, pterygoid; **q**, quadrate; **qj**, quadratojugal; **r.art**, right articular; **r.ect**, right ectopterygoid; **r.exo**, right exoccipital; **r.lacr**, right lacrimal. Scale bars as indicated.
